# The comparisons of expression pattern reveal molecular regulation of fruit metabolites in *S. nigrum* and *S. lycopersicum*

**DOI:** 10.1038/s41598-022-09032-z

**Published:** 2022-03-23

**Authors:** Jung Heo, Woo Young Bang, Jae Cheol Jeong, Sung-Chul Park, Je Min Lee, Sungho Choi, Byounghee Lee, Young Koung Lee, Keunhwa Kim, Soon Ju Park

**Affiliations:** 1 Division of Biological Sciences and Research Institute for Basic Science, Wonkwang University, Iksan, 54538 Republic of Korea; 2 Biological and Genetic Resources Assessment Division, National Institute of Biological Resources, Incheon, 22689 Republic of Korea; 3 Biological Resource Center, Korea Research Institute of Bioscience and Biotechnology (KRIBB), Jeongeup, 56212 Republic of Korea; 4 Department of Horticultural Science, Kyungpook National University, Daegu, 41566 Republic of Korea; 5 Institute of Plasma Technology, Korea Institute of Fusion Energy, 37 Dongjangsan-ro, Gunsan-si, Jeollabuk-do 54004 Republic of Korea

**Keywords:** Plant sciences, Plant biotechnology, Plant development, Secondary metabolism

## Abstract

*Solanum nigrum*, known as black nightshade, is a medicinal plant that contains many beneficial metabolites in its fruit. The molecular mechanisms underlying the synthesis of these metabolites remain uninvestigated due to limited genetic information. Here, we identified 47,470 unigenes of *S. nigrum* from three different tissues by de novo transcriptome assembly, and 78.4% of these genes were functionally annotated. Moreover, gene ontology (GO) analysis using 18,860 differentially expressed genes (DEGs) revealed tissue-specific gene expression regulation. We compared gene expression patterns between *S. nigrum* and tomato (*S. lycopersicum*) in three tissue types. The expression patterns of carotenoid biosynthetic genes were different between the two species. Comparison of the expression patterns of flavonoid biosynthetic genes showed that 9 out of 14 enzyme-coding genes were highly upregulated in the fruit of *S. nigrum*. Using CRISPR-Cas9-mediated gene editing, we knocked out the R2R3-MYB transcription factor *SnAN2* gene, an ortholog of *S. lycopersicum ANTHOCYANIN 2*. The mutants showed yellow/green fruits, suggesting that SnAN2 plays a major role in anthocyanin synthesis in *S. nigrum*. This study revealed the connection between gene expression regulation and corresponding phenotypic differences through comparative analysis between two closely related species and provided genetic resources for *S. nigrum.*

## Introduction

*Solanum nigrum* is a wild black nightshade species belonging to the Solanaceae family, native to Eurasia, and introduced to America, Australasia, and South Africa^1,2^. *S. nigrum* is a common perennial plant found in roadsides, wooded areas, and disturbed habitats. Both ripe fruits and leaves of *S. nigrum* have been used for culinary and traditional medicine purposes in many countries^1^. Previous studies have reported the presence of many beneficial compounds, such as anthocyanidins, glycoproteins, glycoalkaloids, and polyphenolics in *S. nigrum*^3–5^. *S. nigrum* is also rich in amino acids such as arginine, aspartic acid, alanine, isoleucine, L-proline, serine, and valine^6^. Therefore, *S. nigrum* has great potential to be used as a beneficial food source. However, solanine, a toxic steroidal glycoalkaloid (SGA), is found in many parts of *S. nigrum*^7^. The concentration of this alkaloid is the highest in young leaves and green unripe fruits, and the levels decline with maturation^2,8^. Only the ripe fruits or cooked leaves of *S. nigrum* are consumed to avoid toxicity.

Two major classes of fruit secondary metabolites commonly found in the Solanaceae family are carotenoids and flavonoids. Carotenoids are red/yellow pigments that play important roles in photosynthesis and photoprotection, attraction of pollinators and seed dispersers, and biosynthesis of plant hormones such as abscisic acid (ABA) and strigolactones^9^. The metabolic pathways of carotenoids are highly conserved in many plant species and have been extensively studied in tomato (*Solanum lycopersicum*) fruit. During tomato fruit ripening, the expression of enzyme-coding genes, including geranylgeranyl pyrophosphate synthase (*SlGGPS*), phytoene synthase (*SlPSY*), phytoene desaturase (*SlPDS*), zeta-carotene desaturase (*SlZDS*), and carotene isomerase (*SlCRTISO*) are upregulated and are primarily involved in the accumulation of lycopene^9–12^. The expression of these enzyme genes is controlled by environmental (e.g., light, temperature) and internal (e.g., hormones) regulators. Some MADS-box ripening regulators, such as TOMATO AGAMOUS-LIKE1 (TAGL1), Ripening Inhibitor (RIN), FRUITFULL1 (FUL1), and FUL2, and other types of transcription factors (TFs) are involved in this process^13^. Unlike in tomato, little is known about carotenoid metabolism in the fruits of other wild Solanaceae species, including *S. nigrum*.

Flavonoids are important molecules responsible for the color of flowers that attract pollinator animals. Anthocyanins are important flavonoids that play multiple roles in plant development, including protection against biotic and abiotic stresses. The metabolic pathways of anthocyanins are highly conserved in plants, and they are synthesized by a series of enzymes involved in the phenylpropanoid pathway^14^. These biosynthetic enzyme genes are subdivided into two groups: early biosynthetic genes (EBGs: *CHALCONE SYNTHASE* (*CHS*), *CHALCONE ISOMERASE* (*CHI*), and *FLAVANONE 3-HYDROXYLASE* (*F3H*)) and late biosynthetic genes (LBGs: *FLAVONOID 3*′*-HYDROXYLASE* (*F3*′*H*), *FLAVONOID 3*′*5*′*-HYDROXYLASE* (*F3*′*5*′*H*), *DIHYDROFLAVONOL 4-REDUCTASE* (*DFR*), *ANTHOCYANIN SYNTHASE* (*ANS*), and *UDP-GLUCOSE FLAVONOID-3-O-GLUCOSYLTRANSFERASE* (*UFGT*))^14^. In many Solanaceous vegetables, the expression levels of LBGs and anthocyanin content are reported to be positively correlated^14–18^. The expression of anthocyanin biosynthetic genes is regulated mainly by the MYB-bHLH-WD40 (MBW) transcription factor complex. In *S. nigrum*, anthocyanin accumulates in significant quantities only in fully ripened purple fruits, and not in leaves, stems, or green unripe fruit^19^.

Currently, genomes of many members of the Solanaceae species, such as tomato, potato, pepper, and eggplant, have been sequenced, and metabolic enzyme gene expression regulation has been reported to be directly associated with the production of beneficial metabolites. For example, a rare allele in the *TomLoxC* promoter was identified in the tomato pan genome and was selected during domestication. Quantitative trait locus (QTL) mapping and analysis of transgenic plants revealed a role for *TomLoxC* in apocarotenoid production, which contributes to tomato flavor^20^. Furthermore, genome-wide analysis in potato identified 77 genomic loci encoding enzymes involved in starch metabolism, including starch biosynthesis and degradation^21^. Moreover, the chromosome-scale reference genome of black pepper provided insights into piperine biosynthesis, and comparative genomic analyses further revealed specific gene expansions in the glycosyltransferase, cytochrome P450, shikimate hydroxycinnamoyl transferase, lysine decarboxylase, and acyltransferase gene families^22^. Additionally, 121 basic helix–loop–helix (bHLH) transcription factors that are related to anthocyanin biosynthesis in eggplant were identified in the recently released eggplant genome^23^. Unfortunately, only limited genetic resources, such as genome and transcriptome, are available for *S. nigrum*, and studies on metabolic pathways have rarely been conducted.

Here, we profiled the *S. nigrum* transcriptome from mature leaves, reproductive shoot apices, and ripe fruits using the Illumina paired-end platform. The sequencing reads were assembled to create reference unigenes of *S. nigrum*, and we explored the phenotypic differences between *S. nigrum* and *S. lycopersicum* using expression analyses of the unigenes. Moreover, we identified and characterized DEGs and differentially expressed TFs among samples. The results provided an understanding of molecular variations in the metabolic pathways of *S. nigrum* and *S. lycopersicum* and could assist further molecular research of *S. nigrum*.

## Results

### Tissue-specific gene expression profiles of unigenes in *S. nigrum*

**Figure 1:**
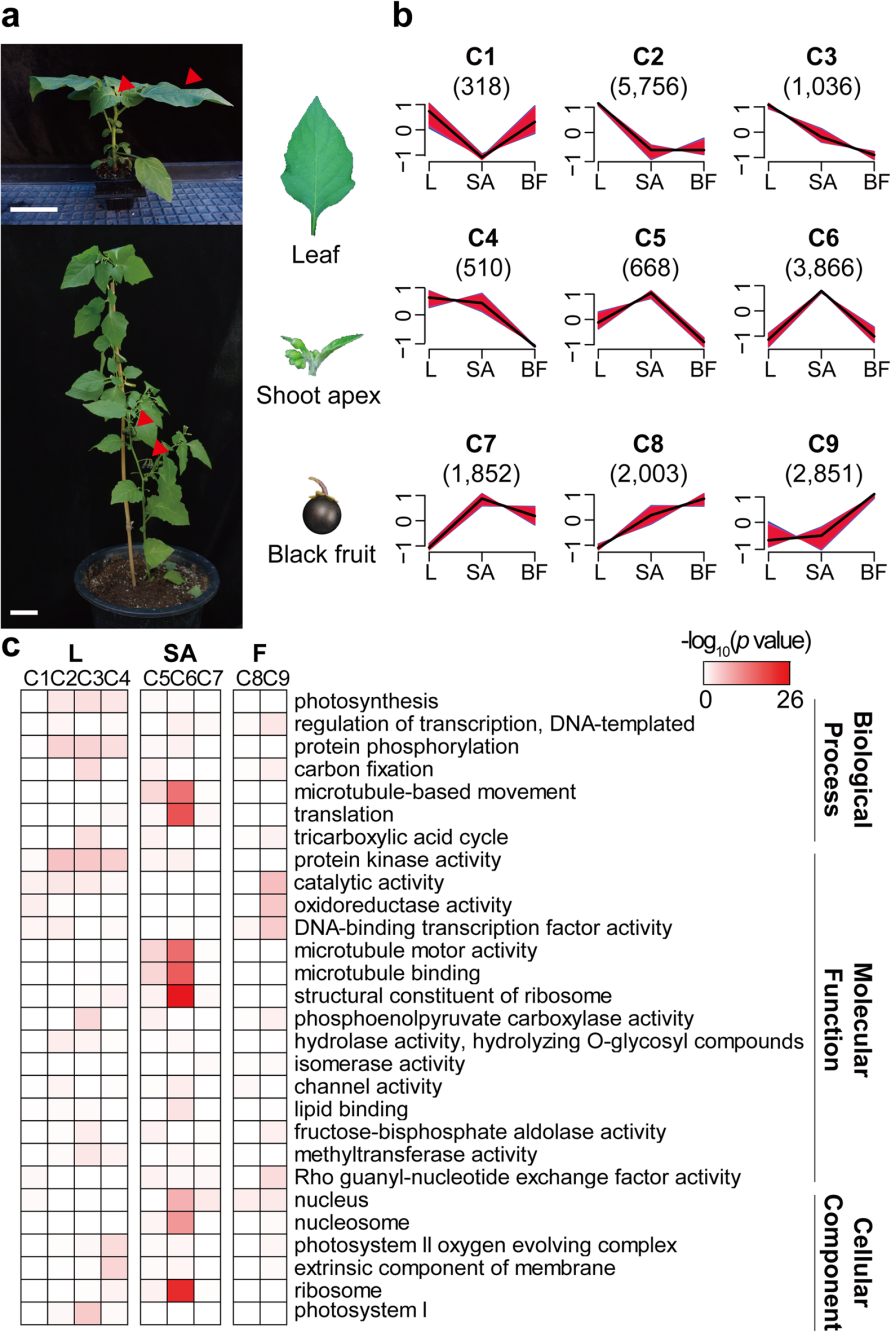
Unigene expression dynamics and enriched GO terms in *S. nigrum* DEGs. (**a**) Representative images for RNA-seq samples in *S. nigrum*. Red arrowheads indicate the collected positions. Scale bars, 6 cm. (**b**) Nine clusters (C1–9) of *S. nigrum* DEGs according to the expression patterns. The numbers in parentheses represent the number of DEGs. X-axis indicates each sample (L, leaf; SA, shoot apex; BF, black fruit) and Y-axis indicates standardized TPM values. (**c**) Enriched GO terms in each cluster. The heat-map color scale represents − log_10_(*p* value) for each GO terms.

To develop a transcriptome of *S. nigrum*, we performed RNA sequencing (RNA-seq) using three different tissue samples: mature leaves, reproductive shoot apices, and mature black fruits (Fig. 1a and Supplementary Table S1), with three biological replicates of each tissue. We primarily focused on the fruit of *S. nigrum,* due to its potential to be used as food. We investigated the shoot apex and the mature fruit which are important for reproductive transition and used leaf, which is the central photosynthetic tissue, as the control. A total of 47,470 unigenes were identified with a transcripts per million (TPM) value greater than 0.3 (Supplementary Table S2). Data quality was validated by correlation assays (Supplementary Fig. S1), and the unigenes were assessed using BUSCO^24^ (Supplementary Fig. S2). Subsequently, 37,223 (78.4%) unigenes were functionally annotated using BLASTP^25^ (Supplementary Tables S3 and S4). The workflow of the entire procedure is summarized in Supplementary Fig. S3. We also noticed that the GC content of the transcripts of *S. nigrum* was 42–43% (Supplementary Table S1), which is in a range similar to that of other GC-poor dicots *Arabidopsis* and tomato^26^. Compared to the GC-rich monocots such as rice (45–50% of GC in transcriptome), *S. nigrum* showed lower GC level, which might imply that *S. nigrum* did not experience any extreme cold or drought conditions during evolution, owing to the low thermal stability^27^.

Based on the normalized read counts of unigenes in all tissues, we identified a total of 18,860 DEGs across the tissue samples using DESeq2^28^ with cut-off criteria: log_2_-fold change ≥ 2, false discovery rate (FDR) < 0.05, and TPM value ≥ 3. The DEGs were clustered into nine clusters (C1–9) according to the expression dynamics in three tissues (Fig. 1b, see “Methods”). Cluster 1–4 (7620 genes) was grouped as a leaf meta-cluster (L) containing genes mainly expressed in the leaf tissue. Genes in cluster 5–7 (6386 genes), grouped as a shoot apex meta-cluster (SA), were highly expressed in the shoot apex. Cluster 8–9 (4854 genes) was grouped as a black fruit meta-cluster (BF), in which gene expression peaked in the black fruit. (Fig. 1b and Supplementary Table S5).

To functionally categorize each cluster, we performed a GO enrichment analysis using topGO^29^. Photosynthesis-related GO terms were highly enriched in the leaf meta-cluster (L, C1–4), consistent with leaf tissue function. Genes related to cell proliferation such as microtubule-based movement and translation were enriched in the shoot apex meta-cluster (SA, C5–7), markedly in C5, and GO terms, including catalytic activity, oxidoreductase activity, and DNA-binding transcription factor activity were highly enriched in the black fruit meta-cluster (BF, C8–9), reflecting tissue-specific functions (Fig. 1c). These data confirmed that gene expression is tightly controlled in a tissue-specific manner in *S. nigrum*.

### Tissue-specific functions of differentially expressed transcription factors

**Figure 2:**
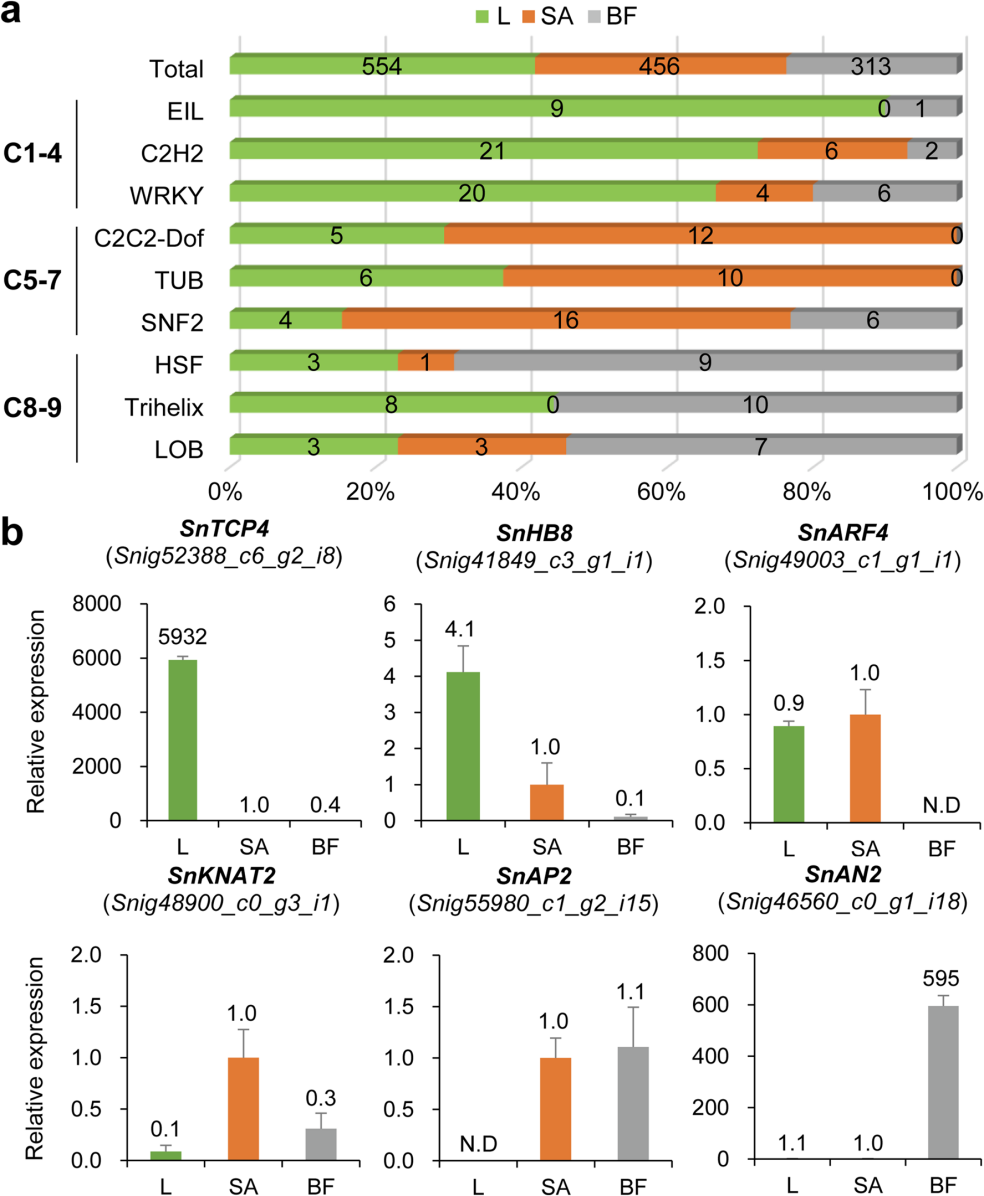
Transcription factor analysis in *S. nigrum* DEGs. (**a**) Top-three enriched transcription factor families in each meta-cluster (L, leaf; SA, shoot apex; BF, black fruit). The gene groups containing a minimum of 10 TFs were tested, and three highest enriched groups are presented for each meta-cluster. All the groups showed hypergeometric *p *value < 0.05. (**b**) qRT-PCR validation of TF DEGs. Expression levels of each gene were normalized with the value of shoot apex and *UBIQUITIN* was used as the endogenous control. Data are shown as mean ± standard deviation: n = 3, biological replicates. N.D, not detected.

To explore the transcriptional regulation that causes differential gene expression profiles in each tissue at the transcriptomic level, we analyzed transcription factors (TFs) in DEGs using the Plant Transcription Factor Database (PlnTFDB)^30^. A total of 1,323 TFs were identified in the DEG set; 554 (41.9%), 456 (34.5%), and 313 (23.6%) TFs of them were included in the leaf meta-cluster (L), shoot apex meta-cluster (SA), and black fruit meta-cluster (BF), respectively (Fig. 2a). To ascertain whether certain specific types of transcription factors play major roles in specific tissues, we categorized all the TF DEGs based on the protein families of PlnTFDB classification (Supplementary Table S6). EIL (Ethylene-Insensitive 3-Like), C2H2-type Zinc finger, and WRKY types of TFs were highly enriched in L; C2C2-Dof (C2C2-type Zinc finger-DNA binding with one finger), TUB (TUBBY), and SNF2 (Sucrose Non Fermenting 2) types were mostly enriched in SA; HSF (Heat Stress Transcription factor), Trihelix, and LOB (Lateral Organ Boundaries) types were enriched in BF compared with the distribution of total TF DEGs (Fig. 2a). These data suggest that tissue-specific control of certain types of transcription factors induces differential expression patterns in downstream networks.

We then validated the expression profiles of some TF DEGs *in planta* using qRT-PCR. *SnTCP4* and *SnHB8* were newly identified *S. nigrum* genes homologous to the *Arabidopsis TCP FAMILY TRANSCRIPTION FACTOR 4* (*TCP4*) and *HOMEOBOX-LEUCINE ZIPPER PROTEIN 8* (*HB8*), respectively, showing leaf tissue-specific expression enrichment. TCP4 regulates leaf cell proliferation^31^ and HB8 functions in leaf vascular formation in *Arabidopsis*^32^. Consistent with the transcriptome data, these two genes were highly upregulated in leaves compared with other tissues, and *SnHB8* showed moderate expression levels in the shoot apex (Fig. 2b). We identified two shoot apex-specific TFs, *SnARF4* and *SnKNAT2*, homologs of *Arabidopsis AUXIN RESPONSE FACTOR 4* (*ARF4*) and *HOMEOBOX PROTEIN KNOTTED-1-LIKE 2* (*KNAT2*), respectively. ARF4 is an auxin signaling component that regulates leaf polarity^33,34^ and promotes flower initiation^35^, showing high expression levels in both the leaf and shoot apex. KNAT2, together with KNAT6, plays an important role in meristem activity and maintenance in *Arabidopsis*^36,37^. The expression pattern of *SnARF4* showed enrichment in the leaf and shoot apex, and *SnKNAT2* was specifically expressed in the shoot apex (Fig. 2b). Furthermore, we tested two black fruit-specific TFs, *SnAP2* and *SnAN2*, homologs of *Arabidopsis APETALA 2* and tomato *ANTHOCYANIN 2* (*SlAN2*), respectively. AP2 plays a central role in the specification of floral organ identity and development of the floral meristem and seeds^38,39^, the expression pattern of which was enriched in both meristem and fruit tissues (Fig. 2b). SlAN2 is a key regulator of anthocyanin biosynthesis majorly expressed in the black fruit of *S. nigrum*, and the tomato fruit turned purple when it was ectopically expressed^40^, (Fig. 2b). Taken together, these data suggest that the functions of well-known transcription factors identified in model organisms are also probably well conserved in *S. nigrum* and transcriptional regulation of the transcription factors possibly cause tissue-specific gene expression profiles.

### Comparison of *S. nigrum* with *S. lycopersicum*

**Figure 3:**
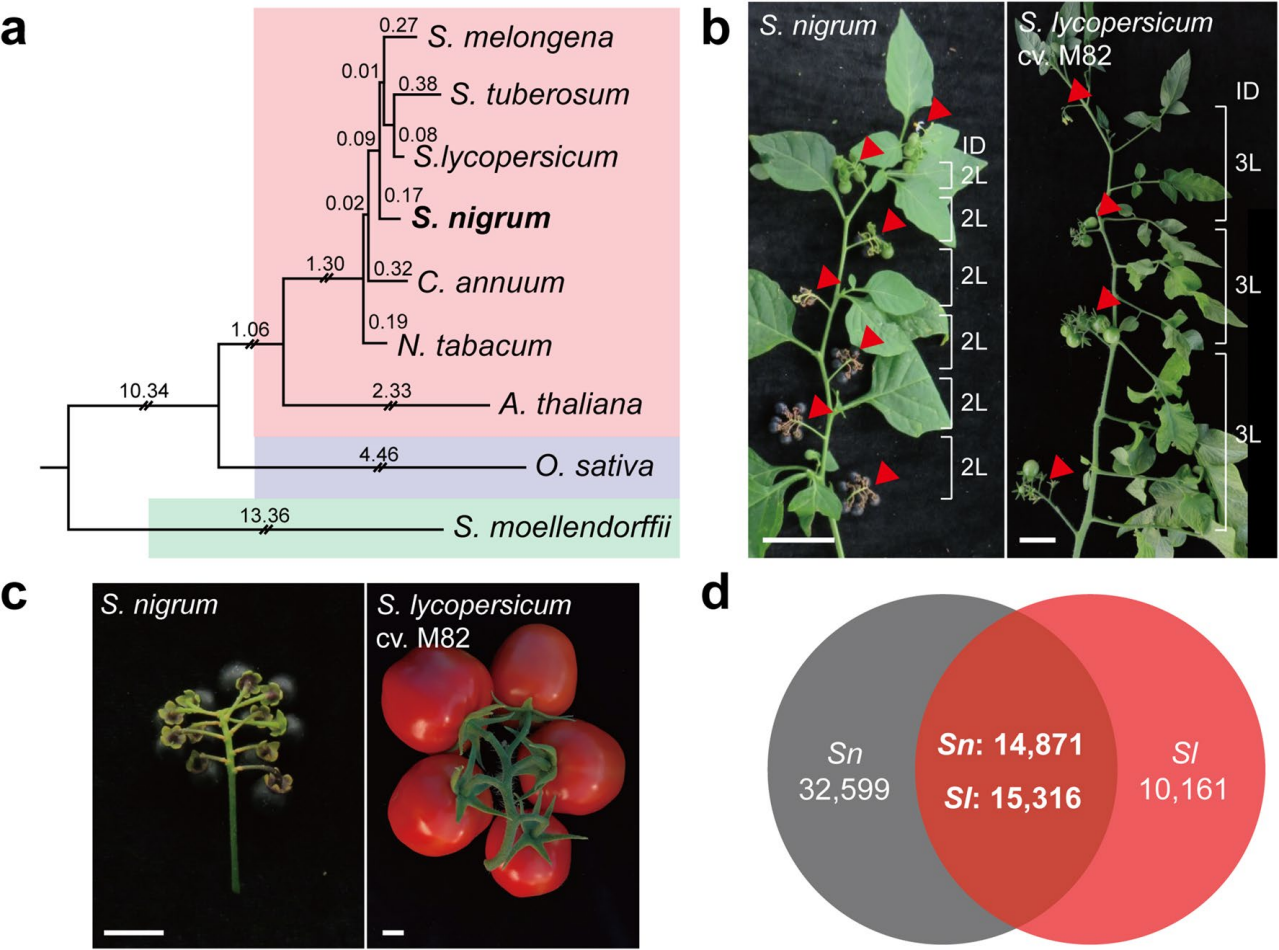
Evolutionary conservation and variation in *S. nigrum* and *S. lycopersicum*. (**a**) Phylogenetic tree constructed using chloroplast proteins of *S. nigrum* with Solanaceae, *Arabidopsis*, rice, and primitive species. Distances in the tree represent percent accepted mutation (PAM) units. Red box, dicot; blue box, monocot; green box, primitive species. (**b**) Comparison of sympodial shoot structure. Red arrowheads indicate inflorescences. Brackets and numbers represent the number of leaves (L) between inflorescences. ID, indeterminate growth. Scale bars, 5 cm. (**c**) Comparison of ripe fruits with inflorescence between *S. nigrum* and *S. lycopersicum*. Scale bars, 1 cm. (**d**) Venn diagram showing orthologs between *S. nigrum* unigenes and *S. lycopersicum-*expressed genes based on hierarchical orthologous groups analysis. *Sn*, *S. nigrum*; *Sl*, *S. lycopersicum*.

As there are limited genetic or genomic resources for the study of *S. nigrum*, we performed a comparative analysis with a closely related species. To identify the plant evolutionarily closest to *S. nigrum*, we constructed a phylogenetic tree with five most representative Solanaceae species. We used the complete chloroplast protein sequences of tomato (*Solanum lycopersicum*), potato (*Solanum tuberosum*), eggplant (*Solanum melongena*), pepper (*Capsicum annuum*), and tobacco (*Nicotiana tabacum*) obtained from GenBank and added the chloroplast protein sequences of *Arabidopsis thaliana* as a reference for the outgroup. We also used two more outgroup controls, *Oriza sativa*, a monocot and *Selaginella moellendorffii*, a lycopodiophyta, to confirm the evolution of the tracheophytes (Supplementary Table S7). As shown in Fig. 3a*. S. lycopersicum* appeared to be the closest relative to *S. nigrum*. Therefore, *S. lycopersicum*, an extensively studied domesticated fruit crop, is a good standard for comparative studies of *S. nigrum*.

In aerial organs, *S. nigrum* and *S. lycopersicum* showed similar indeterminate growth with different sympodial indices (SPIs); two in *S. nigrum* and three in *S. lycopersicum* (cv. M82) (Fig. 3b). Although they showed similar inflorescence structure, the fruit size of *S. nigrum* is much smaller than that of *S. lycopersicum* and is comparable to the fruit of *S. pimpinellifolium,* a wild tomato species^41,42^ (Fig. 3c). The most conspicuous difference between the fruits is their color upon maturation; *S. nigrum* was black, whereas *S. lycopersicum* was red (Fig. 3c). This suggests the accumulation of different metabolites in the fruits of *S. lycopersicum* and *S. nigrum*, possibly due to domestication of *S. lycopersicum* and natural selection in *S. nigrum*. Accordingly, in spite of evolutionary closeness, *S. nigrum* and *S. lycopersicum* show clear differences in morphology, which suggest a significant transcriptomic change between the two species.

To investigate transcriptomic differences, we obtained RNA-seq read data for *S. lycopersicum* from previous studies^43,44^. We then determined the hierarchical orthologous groups (orthogroups) using the OMA standalone^45^ between 47,470 *S. nigrum* unigenes and 25,477 *S. lycopersicum*-expressed genes (see “Methods”). A total of 14,871 of *S. nigrum* genes and 15,316 *S. lycopersicum* genes were identified as orthogroups, which accounted for 60.1% and 31.3% of their total genes, respectively (Fig. 3d). In spite of evolutionary closeness, more than half (68.7%) of the *S. nigrum* unigenes were identified as unlikely to be orthologous to any of the expressed genes in *S. lycopersicum*. This might imply that after divergence, large genomic changes, such as insertion and deletion events, occurred during evolution and domestication, which led to phenotypic variations. Genes included in the orthogroups were further annotated with KEGG Orthology^46^ for the analysis of metabolic pathways (Supplementary Table S8).

### Carotenoid biosynthesis in mature fruit

**Figure 4:**
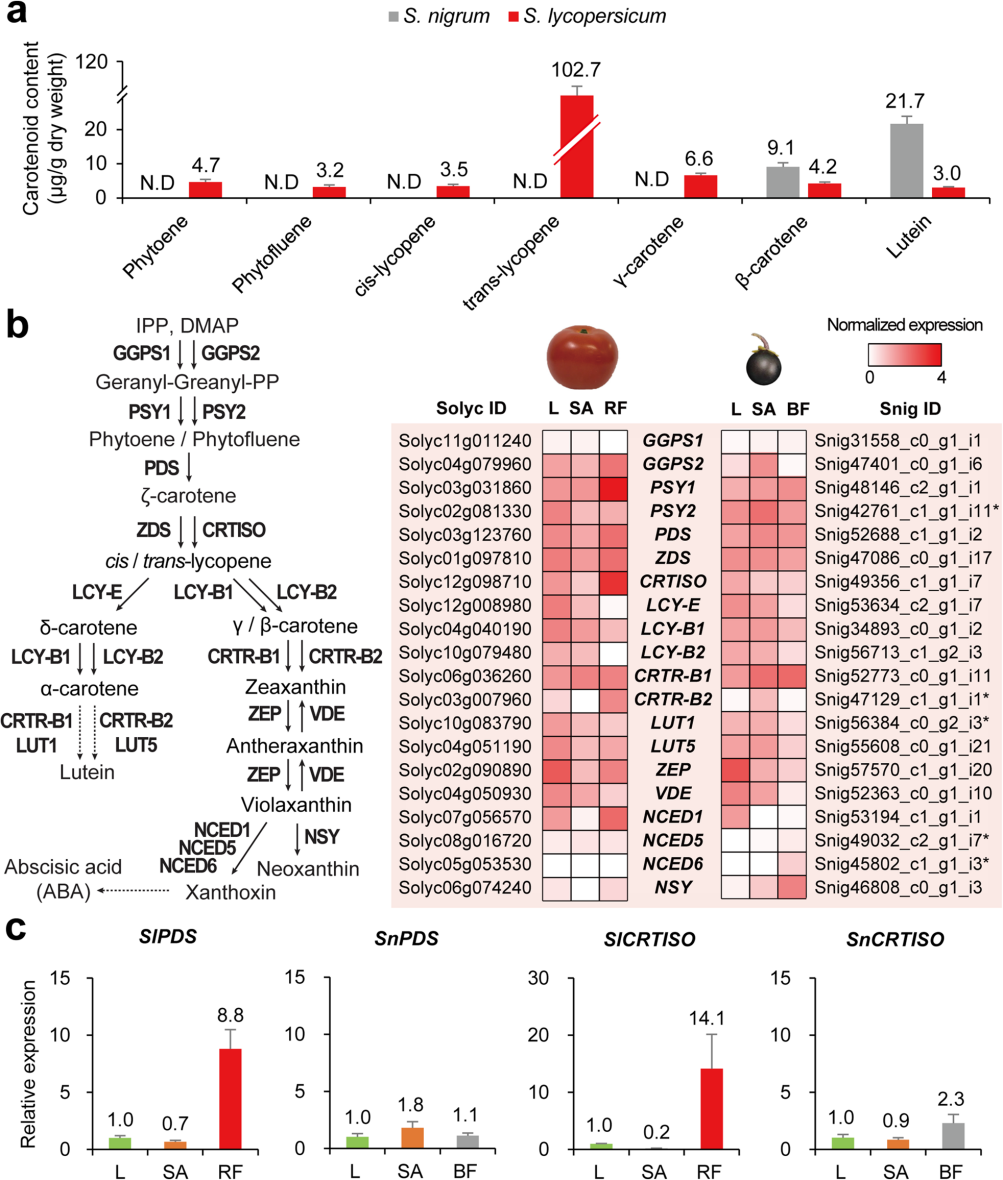
Comparison of metabolites and expression profiles in carotenoid biosynthesis pathway. (**a**) Carotenoid contents in mature fruits of *S. nigrum* and *S. lycopersicum*. Data are shown as mean ± standard deviation: n = 5; five technical replicates, a minimum of 50 fruits were pooled. N.D, not detected. (**b**) Carotenoid biosynthesis pathway and expression profiles of carotenoid biosynthetic genes. Dotted arrows indicate the condensed pathway. *S. nigrum* unigenes with asterisk represent genes with highest homology. The expression is normalized by log_10_(TPM + 1). (**c**) qRT-PCR results for *PDS* and *CRTISO* between the two species. Expression levels of each gene were normalized with the value of leaf and *UBIQUITIN* was used as the endogenous control. Data are shown as mean ± standard deviation: n = 2, pooled samples and two technical replicates. L, leaf; SA, shoot apex; RF, red fruit; BF, black fruit.

We determined the carotenoid content in the ripe fruits of *S. nigrum* and *S. lycopersicum* by high-performance liquid chromatography (HPLC) analysis. In addition to lycopenes and carotenes, the most abundant carotenoids in tomato, we also detected phytoene, phytofluene, and lutein in *S. lycopersicum* (Fig. 4a). However, most of the carotenoids tested were not detected in *S. nigrum*, and only β-carotene and lutein were detected. Interestingly, β-carotene and lutein contents were 2.2-fold and 7.2-fold higher, respectively, in *S. nigrum* than in *S. lycopersicum*. These data indicate that enzyme activities of the carotenoid biosynthesis pathway differ between the two species, resulting in a difference in carotenoid content. It is also possible that the expression of carotenoid biosynthetic enzyme genes is mostly repressed in *S. nigrum*, except for enzymes involved in β-carotene and lutein accumulation.

We revised the carotenoid biosynthesis pathway in tomatoes based on the KEGG pathway (sly00906) and data from previous studies^47,48^, and then tested the expression patterns of 20 genes encoding enzymes in the pathway (Fig. 4b). Of the 20 genes, 15 identified in the orthogroups and an additional five genes which showed the highest homology to *S. lycopersicum* genes were selected by BLASTP. As it is not feasible to directly compare gene expression between two different species, we also prepared the expression profiles of *S. lycopersicum* in three different tissues, as we did for *S. nigrum*. (Supplementary Table S9). The expressions of genes *SlGGPS2*, *SlPSY1*, *SlPSY2*, *SlPDS*, *SlZDS*, and *SlCRTISO* were highly enriched in the red fruit of *S. lycopersicum*, whereas the expressions of the corresponding orthologs were not specifically enriched in the black fruit of *S. nigrum*. This suggests that the carotenoid biosynthetic process is relatively more active in *S. lycopersicum* than in *S. nigrum*. For example, *CRTISO* expression is highly enriched in the red fruit of *S. lycopersicum*, but not in the black fruit of *S. nigrum*, resulting in high accumulation of lycopene only in *S. lycopersicum*. Intriguingly, the expression of *BETA-CAROTENE HYDROXYLASE 1* (*CRTR-B1*) was highly enriched in the black fruit of *S. nigrum* compared with the red fruit of *S. lycopersicum*, which might have caused the elevated levels of lutein in *S. nigrum* (Fig. 4a). *PDS* and *CRTISO* gene expressions were validated by qRT-PCR in both species, and the results showed that expression enrichment was observed only in the red fruit of *S. lycopersicum*, consistent with the in silico data (Fig. 4c).

To further investigate the molecular regulation of carotenoid biosynthesis, we investigated expression patterns of orthologous genes of well-known MADS-box ripening regulators in tomato, *RIN*, *FUL1*, *FUL2*, and *TAGL1*, which are activators of carotenoid biosynthetic genes^13^. Interestingly, *SnRIN*, *SnFUL2* and *SnTAGL1* were as highly enriched in *S. nigrum* fruit as in tomato and thus were included in the BF cluster (Supplementary Table S10). This finding suggests that there might be an antagonistic regulation controlling activators of carotenoid biosynthetic genes in *S. nigrum*, possibly through other BF-enriched transcription regulators. Based on gene expression profiles and HPLC results, we proposed a hypothetical model for the molecular regulation of carotenoid biosynthesis in two species (Supplementary Fig. S4).

### Anthocyanin biosynthesis in mature fruit

**Figure 5:**
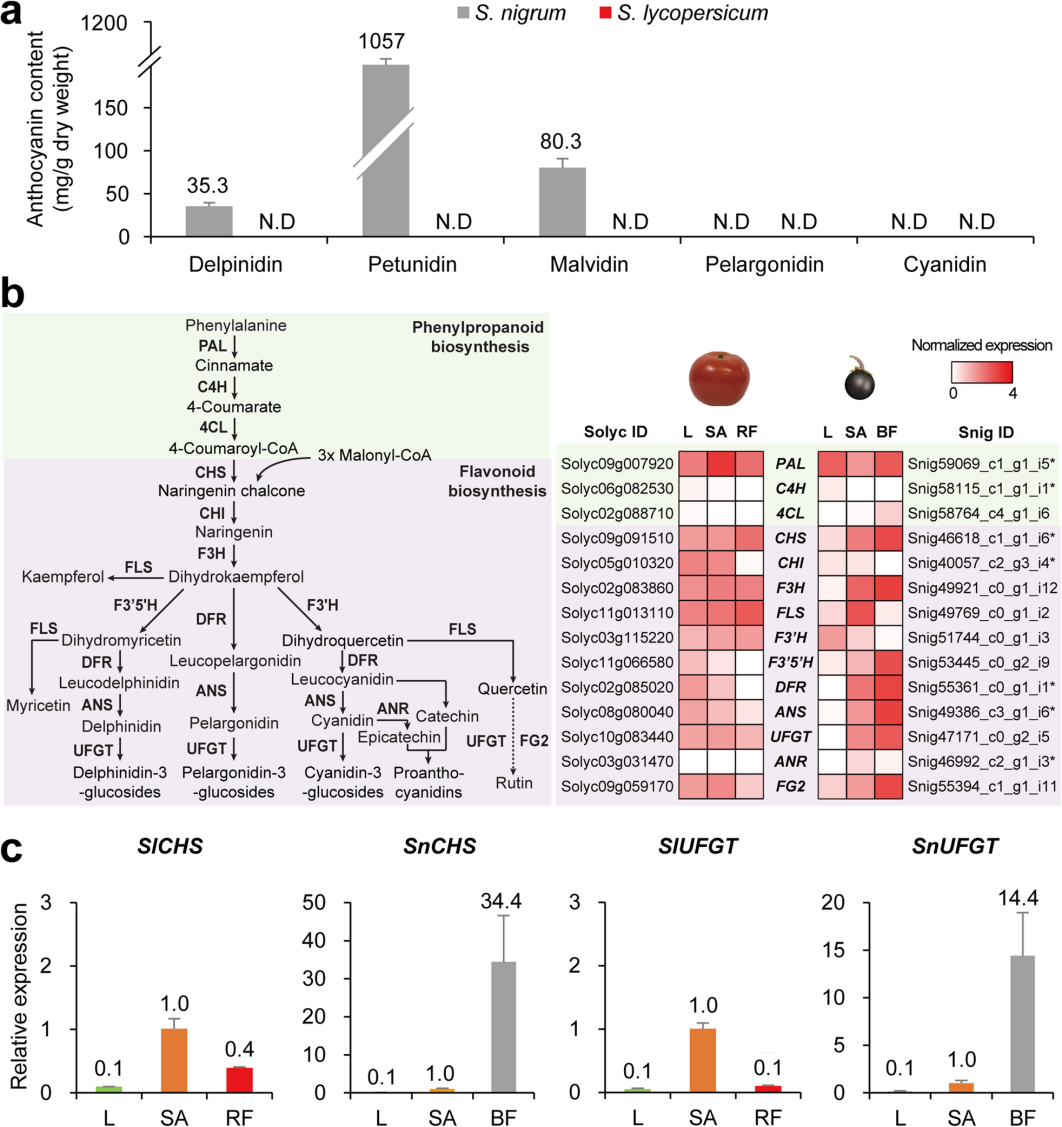
Comparison of metabolites and expression profiles in flavonoid biosynthesis pathway. (**a**) Flavonoid contents in mature fruits of *S. nigrum* and *S. lycopersicum*. Data are shown as mean ± standard deviation: n = 5; five technical replicates, a minimum of 50 fruits were pooled. N.D, not detected. (**b**) Flavonoid biosynthesis pathway and expression profiles of flavonoid biosynthetic genes. Dotted arrow indicates the condensed pathway. *S. nigrum* unigenes with asterisk represent genes with highest homology. The expression is normalized by log_10_(TPM + 1). (**c**) qRT-PCR results for *CHS* and *UFGT* between the two species. Expression levels of each gene were normalized with the value of shoot apex and *UBIQUITIN* was used as the endogenous control. Data are shown as mean ± standard deviation: n = 2, pooled samples and two technical replicates. L, leaf; SA, shoot apex; RF, red fruit; BF, black fruit.

Subsequently, we measured the flavonoid content in the ripe fruits of *S. nigrum* and *S. lycopersicum* by HPLC. Three types of delphinidin-derived flavonoids, delphinidin, petunidin, and malvidin, were detected in *S. nigrum*, but not in *S. lycopersicum* (Fig. 5a). This result suggests that the black color of the fruit of *S. nigrum* is mainly due to the accumulation of flavonoid pigments, consistent with a previous report showing anthocyanin accumulation in *S. nigrum* fruit^49^.

The flavonoid biosynthesis pathway was redrawn based on the KEGG pathway (sly00941) of tomatoes and information from previous studies^50,51^, and expression patterns of 14 enzyme genes were examined. Regarding enzyme genes in *S. nigrum*, 7 out of 14 genes were identified in the orthogroups and an additional seven genes with the highest homology to *S. lycopersicum* genes were selected (Fig. 5b). Although the expression patterns of three enzyme genes involved in phenylpropanoid biosynthesis were comparable in both species, the expression of flavonoid biosynthetic genes was clearly higher in the black fruit of *S. nigrum* than in the red fruit of *S. lycopersicum*. For example, expression of *F3*′*5*′*H*, *DFR*, and *ANS* were not considerably enriched in the red fruit of *S. lycopersicum*, which reflects non-detectable anthocyanin levels in the ripe fruits. On the other hand, high enrichment of the flavonoid biosynthetic gene expression in the black fruit of *S. nigrum* might have caused the accumulation of flavonoid pigments. We could not detect the other kinds of flavonoids, pelargonidins and cyanidins, possibly due to the low sensitivity of our method; otherwise, these pathways could have been deactivated even in *S. nigrum*. qRT-PCR validation showed that the expressions of *SlCHS* and *SlUFGT* were not enriched in the red fruit of *S. lycopersicum*, whereas *SnCHS* and *SnUFGT* expressions were highly enriched in the black fruit of *S. nigrum*, consistent with the in silico data (Fig. 5c).

### Identification of the key transcription factor for anthocyanin biosynthesis in the fruit of *S. nigrum*

**Figure 6:**
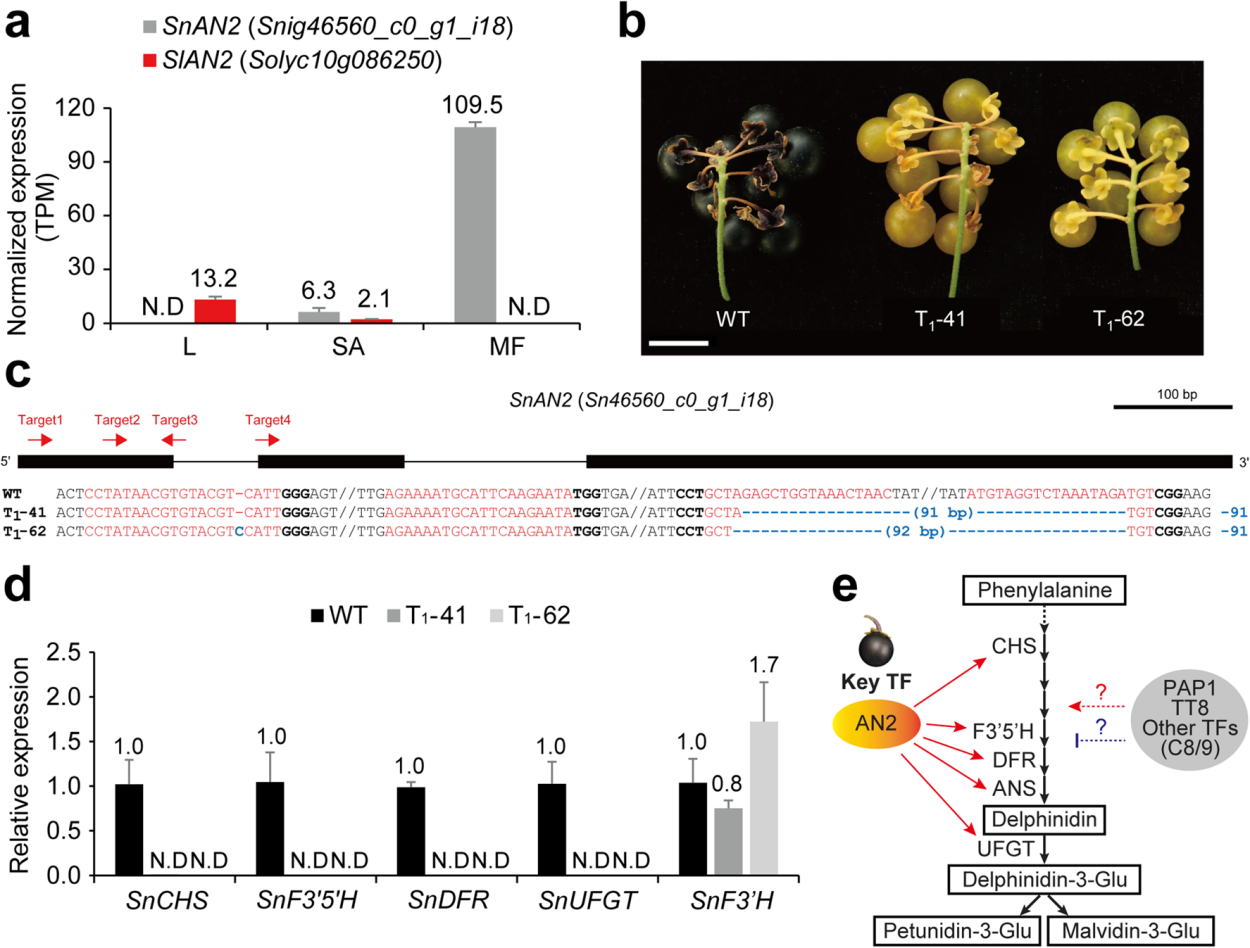
*SnAN2* transcription factor activates flavonoid biosynthetic genes. (**a**) Comparison of *SnAN2* and *SlAN2* expression. L, leaf; SA, shoot apex; MF, mature fruit. Data are shown as mean ± standard deviation. N.D, not detected. (**b**) Representative image of wild-type (WT) and two independent *snan2-cr* mutants for T_1_ generation. Scale bar, 1 cm. (**c**) Gene structure of WT and alleles of the *snan2-cr* mutants. Red and three bold characters, each target (sgRNA) and protospacer adjacent motif (PAM) site. Blue characters, each mutation. (**d**) qRT-PCR results of downstream genes regulated by *SnAN2*. Expression levels of each gene were normalized with the value of WT and *UBIQUITIN* was used as the endogenous control. Data are shown as mean ± standard deviation: n = 4, biological replicates. N.D, not detected. (**e**) Proposed model for the molecular regulation of flavonoid biosynthesis in the fruits of *S. nigrum*. Red arrows (left) indicate the positive regulation shown in this study. Red dashed-arrow and blue dashed-line (right) represent predicted positive and negative regulations. TF, transcription factor. C8/9, Cluster 8 and 9 defined in Fig. 1.

We noticed that the expression of the *SnAN2* gene, an ortholog of *SlAN2*, was significantly enriched in the black fruit of *S. nigrum* (Fig. 2b). *SlAN2* encodes an R2R3-MYB transcription factor, which is sufficient for anthocyanin accumulation when it is ectopically expressed in tomatoes^40^. This prompted us to investigate whether *AN2* gene expression regulation determines fruit color differences between the two species. RNA-seq results showed that, while *SnAN2* gene expression was highly enriched in the black fruit of *S. nigrum*, *SlAN2* expression was not enriched in the red fruit of *S. lycopersicum* (Fig. 6a). Thus, we hypothesized that *SnAN2* plays a major role in anthocyanin biosynthesis in the black fruit of *S. nigrum*. To verify this, we created *SnAN2* knock-out mutants of *S. nigrum* using the CRISPR-Cas9 system. Based on the RNA-seq data, we obtained a full-length genomic sequence of the *SnAN2* gene by PCR and Sanger sequencing, and we designed four single guide RNAs (sgRNAs) targeting the 5’ regions of the gene. Two independent T_1_ transgenic plants were isolated and genotyped, both of which had a large deletion between targets 3 and 4, and one of them had a 1-base pair insertion in the target 1 region (Fig. 6c). Both mutations resulted in premature stop codons and consequent truncated SnAN2 proteins, the MYB domains of which were fully disrupted, implying possible null mutants (Supplementary Fig. S5). As a result, both mutant plants failed to properly synthesize anthocyanin; thus, mature fruits turned yellow/green in color (Fig. 6b). Expression of anthocyanin biosynthetic genes was tested by qRT-PCR. *SnCHS*, *SnF3*′*5*′*H*, *SnDFR*, and *SnUFGT* expressions were decreased in the two mutant lines compared with the wild-type, whereas *SnF3’H* expression was not influenced (Fig. 6d). These data suggested that SnAN2 is mainly required for the expression of genes encoding anthocyanin biosynthesis enzymes and transcriptional induction of *SnAN2* is essential for anthocyanin production during ripening of fruits in *S. nigrum* (Fig. 6e).

In addition to *AN2*, a number of transcription regulators were characterized in plant anthocyanin biosynthesis pathways^52^. We found that the expressions of orthologous genes of *Production of Anthocyanin Pigment 1* (*PAP1*), another MYB transcription factor known as an activator for anthocyanin biosynthetic genes in *Arabidopsis*, and *Transparent Testa 8* (*TT8*), a bHLH transcription activator for anthocyanin biosynthetic genes in tobacco, were enriched in the BF-cluster in *S. nigrum*. Alternatively, an ortholog of a homeodomain-leucine zipper transcription factor, *GLABRA2*, a potential repressor for anthocyanin production, was also enriched in the fruit of *S. nigrum* (Supplementary Table S11). This implies that the orchestrated functions of enriched TFs finely regulate anthocyanin biosynthetic gene expressions in the fruits of *S. nigrum* (Fig. 6e).

### Sugar contents in mature fruit

In addition to the pigment contents, we also measured the levels of carbohydrates, including fructose, glucose, sucrose, maltose, and lactose, which are primary metabolites. In ripe fruits, only the monosaccharides fructose and glucose were detected in both species, and the levels were 3.9- and 4.4-fold higher, respectively, in *S. nigrum* than in *S. lycopersicum* (Supplementary Fig. S6). The sugar metabolism pathway of *S. nigrum* was drawn based on the KEGG pathway (sly00500) of tomatoes and information from previous studies^53,54^ (Supplementary Fig. S6). A total of 29 sugar metabolic genes of *S. nigrum* were identified in the orthogroups and 23 best-hit homologs were also found using BLASTP. Some genes encoding Sucrose Synthase (Susy) and some genes encoding cell wall invertases showed high enrichment of expression in the black fruit of *S. nigrum* and only moderate enrichment in the red fruit of *S. lycopersicum* (Supplementary Fig. S6). This might have caused the difference in sugar levels between the two species.

## Discussion

Many wild crop species are utilized as food sources and in medicinal applications worldwide. Although domestication and molecular breeding of these wild plants are important for improving crop yield and usage, these are not easily achieved due to lack of genetic information. Therefore, the first step would be to obtain genetic resources for the domestication of wild species. *S. nigrum* has great potential as a medicinal plant and is used in many countries^1,2^. In this study, we identified 47,470 unigenes in *S. nigrum* by de novo transcriptome assembly from three tissue samples. In total, 78.4% of the unigenes were functionally annotated and DEGs in the tissue samples were classified by expression dynamics (Fig. 1). These data could be used as valuable genetic information resources for *S. nigrum*. We also performed a comparative analysis using *S. lycopersicum*, a widely used domesticated crop. This information might help in the de novo domestication of wild black nightshade species. For example, tomato domestication genes, such as *SELF PRUNING* (*SP*), which is important for the development of shoot architecture^55^, and *CLAVATA3* (*CLV3*), which is a main regulator of tomato fruit size^56^, were found in the orthologous gene groups of *S. nigrum*, and the expression regulation of these genes in *S. nigrum* was similar to that in *S. lycopersicum* (Supplementary Tables S3, S8, and S9). Using CRISPR-mediated editing of these domestication genes, crop yield and usage of *S. nigrum* could be enhanced.

*Solanum lycopersicum* and *S. nigrum* are mostly similar in terms of shoot architecture. However, one of the notable differences in the aerial organs is the shape of leaves. *S. lycopersicum* has compound leaves, and *S. nigrum* has simple leaves (Fig. 3b). Many factors determining the leaf architecture were isolated in the orthologs of unigene sets (Supplementary Table S8). For example, an ortholog of Class I KNOX (KNOXI), knotted1-like homeobox transcription factors, may be required for the initiation of compound leaf development^36,57^. In addition, an ortholog of LANCEOLATE (LA), the CINCINNATA (CIN)-like TCP transcription factor, may regulate the activity of the leaf marginal blastozone^58–60^, and NO APICAL MERISTEM (NAM)/CUP-SHAPED COTYLEDON (CUC) proteins, which control the organ boundary, may also play a role in leaf development by suppressing auxin signaling between laminar regions^61^. The difference in leaf structure between *S. nigrum* and *S. lycopersicum* may also provide a clue about evolutionary divergence, and further analyses regarding expression patterns of relevant genes and evolutionary conserveness in these species are required.

Comparative transcriptome analysis of *S. nigrum* and *S. lycopersicum* was performed using tissue-level expression profiling as the direct comparison of expression levels might be misleading. We used three representative tissues from both species and compared the expression patterns of genes of enzymes involved in metabolic pathways. We systematically defined the gene expression profiles of enzymes involved in carotenoid biosynthesis (Fig. 4), anthocyanin biosynthesis (Fig. 5), and sugar metabolism (Supplementary Fig. S6) in *S. nigrum* and *S. lycopersicum* and found key enzyme genes that showed differential expression patterns, which possibly result in phenotype differences. This suggested that comparative analysis using a tissue-level transcriptome assay could successfully signify the phenotypic variations between two different species. However, there might be some limitations, such as missing DEGs and homologs, because of the lack of whole genome information. To compare gene diversification and variations in gene expression regulation more precisely in two closely related species, genomic comparison at the whole-genome level should be performed.

We explored the differences in metabolite contents in ripe fruits of *S. nigrum* and *S. lycopersicum* by HPLC analysis and comparative expression profiling of enzyme genes. We found that the fruits of *S. nigrum* contain higher levels of many metabolites beneficial for human health, such as β-carotene, lutein, and anthocyanin antioxidants, when compared with tomato fruits. Therefore, the fruits of *S. nigrum* could be utilized as dietary supplements or as edible fruits like tomatoes. To achieve this, toxic compounds, such as α-solanine and α-chaconine, need to be removed. Although it is known that these compounds are not detectable in fully ripe fruit of *S. nigrum*, some of the maturing fruits can contain them^7^. Therefore, we briefly investigated the steroidal glycoalkaloid (SGA) biosynthesis pathway in *S. nigrum*. Based on the KEGG pathway (map01066) and information from a previous study^62^, a total of 14 SGA biosynthesis genes of *S. nigrum* identified in the orthogroups and best-hit homologs were examined (Supplementary Fig. S7). Interestingly, the expression patterns of SGA biosynthesis genes were mostly similar in *S. nigrum* and *S. lycopersicum*. *STEROL ALKALOID GLYCOSYLTRANSFERASE* (*SGT*) family genes, which encode enzymes that produce α-solanine and α-chaconine, were weakly expressed in the fruits of *S. nigrum* and *S. lycopersicum*, possibly indicating that the fruits of *S. nigrum* contain relatively less toxic SGA contents than other black nightshade species^7^. Detailed analysis of SGA synthesis in *S. nigrum* is required. For examples, tomato *GAME4* (*GLYCOALKALOID METABOLISM 4*) has been reported to play a key role in the biosynthesis of SGA^63^. Therefore, the enzyme activity of *SnGAME4* could be modified to effectively reduce SGA level. We also noticed that SGA levels decline as fruits mature, and controlling fruit ripening could be another strategy for reducing it. In tomato, a *self-pruning* (*sp*) mutant showed determinate shoot growth, and this mutation can be used for identical fruit maturation^55,64^. This suggested that modifying *SnSP* gene activity can facilitate the synchronization of fruit maturation and simultaneous ripe fruit harvest. The plant hormone ethylene plays a key role in fruit ripening^65^, and molecular control of biosynthesis and signaling of ethylene can also facilitate fruit maturation control.

Sugar content was higher in the fruits of *S. nigrum* than in *S. lycopersicum* (Supplementary Fig. S6). *Lycopersicum Invertase5* (*LIN5*), a cell wall invertase gene, has been reported to be the key enzyme influencing sugar uptake in tomato fruit. *LIN5-*RNAi knockdown transformants were characterized by reduced transpirational water loss in mature fruits accompanied by thickened cuticles^66^. Therefore, upregulated cell wall invertases presumably help in the uptake of more sugars into the *S. nigrum* fruit than in the fruit of *S. lycopersicum*. Further analyses are required.

In conclusion, we successfully generated transcriptomic information and data about the unigenes of *S. nigrum* for extensive molecular studies in the future. Through comparative analysis with tomato, which is one of the best characterized Solanaceae species at the genomic and molecular level, we were able to identify numerous important factors regulating the growth and development of *S. nigrum* and useful primary and secondary metabolites produced in the fruits of *S. nigrum*. Further, we tried to edit a gene involved in anthocyanin biosynthesis based on transcriptomic information, through which control anthocyanin accumulation in the fruits was controlled. This implies that we could rapidly domesticate *S. nigrum* by editing evolutionarily conserved genes related to plant development and production of useful metabolites.

## Methods

### Permission

No specific permits were required for growing *S. nigrum* plants at the greenhouse in Wonkwang University, Iksan, Republic of Korea. Transgenic and *SnAN2* editing mutants were grown on LMO growth room (LML16-1201) permitted by National Research Safety Headquarters in Republic of Korea. All the methods complied with relevant institutional, national, and international guidelines and legislation for scientific research.

### Plant materials and growth conditions

*S. nigrum* seeds (NIBRGR0000189638) were collected and provided by NIBR, Incheon, Republic of Korea. Plants were grown in a greenhouse under long-day conditions (16 h light, 26–28 °C/8 h dark, 18–20 °C; 40–60% relative humidity) supplemented with artificial light from 200 W halogen lamps at Wonkwang University, Iksan, Republic of Korea. Seeds were directly sown on the soil in 96-cell plastic flats, and seedlings were grown for four weeks on the flats. For harvesting fruits, some of the seedlings were transplanted to pots in the greenhouse. All the plants were grown under drip irrigation and standard fertilizer regimes.

### RNA sequencing

Mature leaves that were fourth from the bottom, except for the cotyledon and shoot apices (containing one leaf primordium), of the reproductive stage were harvested 30 days after sowing. A minimum of eight shoot apex samples were pooled. Black fruits were harvested when the fruits were the most mature. A minimum of 50 black fruit samples were pooled. All samples were harvested with three biological replicates between 10 and 11 a.m. The samples were immediately frozen in liquid nitrogen and stored at − 80 °C.

Total RNA of the samples was extracted using the RNeasy® Plant Mini Kit (QIAGEN, Valencia, CA, USA) for leaf and shoot apex, and the Ribospin™ Seed/Fruit Kit (GeneAll Biotechnologies, Republic of Korea) for black fruit, including on-column DNase treatment using the RNase-Free DNase set kit (QIAGEN), according to the manufacturers’ instructions. The extracted total RNA samples were analyzed for concentration and quality using the ND-1000 system (NanoDrop Technologies, Wilmington, DE, USA) and the 2100 Bioanalyzer (Agilent Technologies, Palo Alto, CA, USA). A total of 1 µg of RNA was used for library construction, with the NEBNext® mRNA Library Prep Master Mix for Illumina® Kit (New England Biolabs, Beverly, MA, USA) for leaf and shoot apex and the TruSeq Stranded mRNA Library Prep Kit (Illumina, San Diego, CA, USA) for black fruit, according to the manufacturers’ instructions. Libraries of 70–370 bp (mean 160 bp) insert size were constructed and sequenced using the Illumina HiSeq 2500 (leaf and shoot apex) and the NovaSeq 6000 (black fruit) to generate 101-bp paired-end reads.

### De novo transcriptome assembly and functional annotation

The raw reads were checked for quality using FastQC v0.11.7 (https://www.bioinformatics.babraham.ac.uk/projects/fastqc/) and preprocessed to remove adaptor sequences and low-quality reads using Trimmomatic v0.36^67^ with the following parameters: ILLUMINACLIP:TruSeq3-PE-2.fa:2:30:10, LEADING:20, TRAILING:20, MINLEN:25, and phred33. To build a suitable set of reference contigs, a total of 586,099,338 clean reads were pooled and assembled using Trinity v2.4.0^68^ with the following parameter: min_contig_length 300. Further clustering was then performed using CD-HIT-EST v4.6^69^ with a 95% similarity parameter to obtain non-redundant transcripts. To identify coding regions within transcripts, the longest open reading frames were predicted using TransDecoder v3.0.1 (https://github.com/TransDecoder). To obtain gene expression profiles, the clean reads were aligned to coding sequences using Bowtie v2.2.6^70^, and the abundance of each transcript was estimated and normalized to transcripts per million (TPM) values using RSEM v1.2.31^71^. Genes showing less than 0.3 TPM values were removed, and these sequences were defined as *S. nigrum* unigenes. To validate the expression profiles, correlation analysis was performed using corrplot R package v0.84 (https://github.com/taiyun/corrplot) and the unigenes were assessed using BUSCO v3.1.0^24^ with an embryophyta (version, odb10) lineage dataset (Supplementary Fig. S1 and S2).

To predict the functions of the unigenes, gene functions were annotated using BLASTP v2.9.0 search^25^ based on Araport11, TrEMBL (Ensembl Plants), and Swiss-Prot (Ensembl Plants) with the following parameters: e-value 1e-10, outfmt 6, num_alignments 1, and max_hsps 1. Gene functions were also annotated with GO and Pfam using InterProScan v5.31–70.0^72^. Moreover, KEGG Orthology was annotated using GHOSTZ search and single-directional best hit (SBH) method with the *S. lycopersicum* gene set in the KAAS v2.1 web tool (https://www.genome.jp/kegg/kaas/).

### DEG and transcription factor analysis

To identify differentially expressed genes (DEGs) among leaves, shoot apices, and black fruits, the expression profiles were filtered using DESeq2 v1.26.0^28^ with the following criteria: log_2_-fold change ≥ 2, FDR < 0.05, and TPM values ≥ 3. The DEGs were clustered based on a fuzzy *c*-means algorithm using Mfuzz R package v2.44.0^73^. To decipher the biological functions of each cluster, GO enrichment analysis was performed using topGO R package v2.36.0^29^ with the weight01 algorithm and Fisher's exact test. Enriched GO terms with a *p* value < 0.01 were selected.

To identify differentially expressed transcription factors (TFs) and determine their roles, plant-specific TFs were used from PlnTFDB (http://plntfdb.bio.uni-potsdam.de/v3.0/) and BLASTP search was performed with the following parameters: e-value 1e-10, outfmt 6, num_alignments 1, and max_hsps 1. The putative TFs were filtered by % identity ≥ 50 and those having Pfam domains.

### Ortholog analysis

To identify the plant evolutionarily closest to *S. nigrum*, ortholog analysis was performed using OMA standalone v2.2.0^45^ with chloroplast protein sequences and a phylogenetic tree was constructed using MEGA X^74^. To investigate transcriptomic differences, we obtained RNA-seq read data of *S. lycopersicum* from NCBI Sequence Read Archive: SRP010775^43^, leaf and red fruit; PRJNA343677^44^, TM, FM, SIM, and SYM, ftp://ftp.solgenomics.net/transcript_sequences/by_species/Solanum_lycopersicum/libraries/illumina/LippmanZ). Raw reads were preprocessed, and then the *S. lycopersicum*-expressed genes were defined using the same process utilized in *S. nigrum*. To identify hierarchical orthologous groups between *S. nigrum* and *S. lycopersicum*, OMA standalone was performed with protein sequences of *S. nigrum* unigenes and *S. lycopersicum*-expressed genes.

### qRT-PCR validation

To determine the reliability of the RNA-seq data, qRT-PCR was performed on the same RNA pools used for RNA-seq. A total of 1 µg of RNA was used for cDNA construction using the ReverTra Ace® -α- Kit (TOYOBO, Osaka, Japan), according to the manufacturers’ instructions. qRT-PCR was performed using the StepOnePlus™ Real-Time PCR System (Thermo Fisher, Waltham, MA, USA) with iQ™ SYBR® Green Supermix (Bio-Rad, Hercules, CA, USA). The PCR reaction conditions were: 95 °C for 3 min, followed by 40 cycles of 95 °C for 15 s, 58 °C for 30 s, and 72 °C for 30 s; melt curve stage: 95 °C for 15 s, 55 °C for 15 s, and then increase up to 95 °C by 1.0 °C. Relative gene expression was calculated based on the 2^−∆∆*CT*^ method^75^. The primer sequences used are listed in Supplementary Table S12.

### HPLC analysis

To determine the anthocyanin content, anthocyanins were extracted from 0.2 g of finely ground black and red fruits of *S. nigrum* and *S. lycopersicum*, respectively. Experiments were performed as previously described with minor modifications^76^. Briefly, lyophilized samples were extracted with 1 ml of acidic methanol containing 1% HCl (v/v) for 18 h at room temperature (25 ± 2 °C) with moderate shaking. Subsequently, 500 μl of the supernatant was mixed with 500 μl of HPLC-grade H_2_O and 300 μl of chloroform to remove carotenoids. The water–methanol phase extracts (100 μl) were hydrolyzed. The samples were added to 900 μl of solvent [95:5 (v/v), n-butanol (100%):HCl (36%)], and the mixture was boiled for 2 h to release the core anthocyanidins. Then, the samples were dried in a speed vacuum at room temperature, and the residues were dissolved in 100 μl of 0.1% HCl–methanol solvent. The core anthocyanidins were identified in the supernatant by HPLC analysis using an Agilent 1260 Infinity II system (Agilent technologies, Santa Clara, CA, USA) with a Gemini column (5 µm C18 110A, 120 × 4.6 mm) sourced from Phenomenex (Torrance, CA, USA). All chromatograms were recorded at 520 nm. Pelargonin, delphinidin, cyanidin, petunidin, peonidin-3-O-glucoside (hydrolyzed), and malvidin (Sigma-Aldrich, USA) were used as standards for identification.

To determine the carotenoid content, approximately 0.1 g of frozen pericarp powder from ripe *S. nigrum* and *S. lycopersicum* fruits was used for carotenoid extraction, as previously described^77^. Extracted carotenoids were analyzed using a 1260 Infinity HPLC system (Agilent Technologies, Inc., Santa Clara, CA, USA) equipped with a YMC Carotenoid C30 S-5 column (4.6 × 250 mm). Each carotenoid was identified based on the absorption maxima and spectrum^78^.

To determine the sugar content, sugars were extracted from 0.5 g of finely ground black and red fruits of *S. nigrum* and *S. lycopersicum,* according to the Korean Food Standards Codex method (http://www.foodsafetykorea.go.kr/foodcode). Briefly, lyophilized samples were extracted with 30 ml of ethanol and mixed well using a reciprocating shaker for 15 min at room temperature at 200 rpm. Subsequently, the mixtures were sonicated in a water bath at 80 °C for 25 min. After cooling at room temperature, the mixtures were filtered using 0.2-μm syringe filters. The sugars were identified from the filtered mixtures using HPLC analysis using an Agilent 1260 Infinity II system (Agilent technologies, Santa Clara, CA, USA) with Imtakt Unison UK-Amino column (3 µm, 250 × 3.0 mm). Fructose, glucose, sucrose, maltose, and lactose (Sigma-Aldrich, USA) were used as standards for identification.

### CRISPR-Cas9 mutagenesis and plant transformation

CRISPR-Cas9 mutagenesis of *S. nigrum* was performed as described previously^79^. Briefly, gRNAs were designed using the CRISPRdirect web tool (https://crispr.dbcls.jp/), and binary vectors were built through golden gate cloning as described^80^. The final binary plasmids were introduced into *S. nigrum* cotyledons by *Agrobacterium tumefaciens*-mediated transformation as described previously^81^. Transplantation of transgenic plants and genotyping of CRISPR-generated mutations were performed as previously described^79^. The gRNA and primer sequences used are listed in Supplementary Table S12.

## Supplementary Information

Supplementary Information 1.

Supplementary Information 2.

## Data Availability

All datasets supporting the conclusions of this article are included in the article and supplementary files. The RNA-seq raw reads were deposited in the NCBI Sequence Read Archive (SRA) under BioProject accession PRJNA768612.

## References

[1] Jabamalairaj A, Priatama RA, Heo J, Park SJ (2019). Medicinal metabolites with common biosynthetic pathways in *Solanum nigrum*. Plant Biotechnol. Rep..

[2] Edmonds JM, Chweya JA (1997). Black Nightshades: Solanum nigrum L. and Related Species. Promoting the Conservation and Use of Underutilized and Neglected Crops.

[3] Lee KR (2004). Glycoalkaloids and metabolites inhibit the growth of human colon (HT29) and liver (HepG2) cancer cells. J. Agric. Food Chem..

[4] Sikdar M, Dutta U (2008). Traditional phytotherapy among the Nath people of Assam. Stud. Ethno-Med..

[5] Ravi V, Saleem TSM, Patel SS, Raamamurthy J, Gauthaman K (2009). Anti-inflammatory effect of methanolic extract of *Solanum nigrum* Linn Berries. Int. J. Appl. Res. Nat. Prod..

[6] Ganguly P, Gupta AK, Majumder UK, Ghosal S (2009). The chemistry behind the toxicity of black nightshade, *Solanum nigrum* and the remedy. Pharmacologyonline.

[7] Sammani A, Shammaa E, Chehna F (2013). Qualitative and quantitative steroidal alkaloids of solanum species distributed widely in Syria by TLC and HPLC. Int. J. Pharm. Sci. Rev. Res..

[8] Eltayeb EA, Al-Ansari AS, Roddick JG (1997). Changes in the steroidal alkaloid solasodine during development of *Solanum nigrum* and *Solanum incanum*. Phytochemistry.

[9] Liu L, Shao Z, Zhang M, Wang Q (2015). Regulation of carotenoid metabolism in tomato. Mol. Plant.

[10] Giuliano G, Bartley GE, Scolnik PA (1993). Regulation of carotenoid biosynthesis during tomato development. Plant Cell.

[11] Fraser PD, Truesdale MR, Bird CR, Schuch W, Bramley PM (1994). Carotenoid biosynthesis during tomato fruit development: evidence for tissue-specific gene expression. Plant Physiol.

[12] Pecker I, Gabbay R, Cunningham FX, Hirschberg J (1996). Cloning and characterization of the cDNA for lycopene beta-cyclase from tomato reveals decrease in its expression during fruit ripening. Plant Mol. Biol..

[13] Stanley L, Yuan YW (2019). Transcriptional regulation of carotenoid biosynthesis in plants: So many regulators, so little consensus. Front. Plant Sci..

[14] Liu Y (2018). Anthocyanin biosynthesis and degradation mechanisms in Solanaceous vegetables: A review. Front. Chem..

[15] Borovsky Y, Oren-Shamir M, Ovadia R, De Jong W, Paran I (2004). The A locus that controls anthocyanin accumulation in pepper encodes a MYB transcription factor homologous to Anthocyanin2 of Petunia. Theor. Appl. Genet..

[16] André CM (2009). Influence of environment and genotype on polyphenol compounds and in vitro antioxidant capacity of native Andean potatoes (*Solanum tuberosum* L.). J. Food Compos. Anal..

[17] Povero G, Gonzali S, Bassolino L, Mazzucato A, Perata P (2011). Transcriptional analysis in high-anthocyanin tomatoes reveals synergistic effect of Aft and atv genes. J. Plant Physiol..

[18] Aza-González C, Herrera-Isidrón L, Núñez-Palenius HG, Martínez De La Vega O, Ochoa-Alejo N (2013). Anthocyanin accumulation and expression analysis of biosynthesis-related genes during chili pepper fruit development. Biol. Plant..

[19] Huang HC, Syu KY, Lin JK (2010). Chemical composition of *Solanum nigrum* linn extract and induction of autophagy by leaf water extract and its major flavonoids in AU565 breast cancer cells. J. Agric. Food Chem..

[20] Gao L (2019). The tomato pan-genome uncovers new genes and a rare allele regulating fruit flavor. Nat. Genet..

[21] Van Harsselaar JK, Lorenz J, Senning M, Sonnewald U, Sonnewald S (2017). Genome-wide analysis of starch metabolism genes in potato (*Solanum tuberosum* L.). BMC Genomics.

[22] Hu L (2019). The chromosome-scale reference genome of black pepper provides insight into piperine biosynthesis. Nat. Commun..

[23] Tian S, Li L, Wei M, Yang F (2019). Genome-wide analysis of basic helix–loop–helix superfamily members related to anthocyanin biosynthesis in eggplant (*Solanum melongena* L.). PeerJ.

[24] Simão FA, Waterhouse RM, Ioannidis P, Kriventseva EV, Zdobnov EM (2015). BUSCO: Assessing genome assembly and annotation completeness with single-copy orthologs. Bioinformatics.

[25] Altschul SF, Gish W, Miller W, Myers EW, Lipman DJ (1990). Basic local alignment search tool. J. Mol. Biol..

[26] Kotwal S (2016). De novo transcriptome analysis of medicinally important plantago ovata using RNA-seq. PLoS ONE.

[27] Šmarda P (2014). Ecological and evolutionary significance of genomic GC content diversity in monocots. Proc. Natl. Acad. Sci. USA.

[28] Love MI, Huber W, Anders S (2014). Moderated estimation of fold change and dispersion for RNA-seq data with DESeq2. Genome Biol..

[29] Alexa A, Rahnenführer J, Lengauer T (2006). Improved scoring of functional groups from gene expression data by decorrelating GO graph structure. Bioinformatics.

[30] Pérez-Rodríguez P (2009). PlnTFDB: Updated content and new features of the plant transcription factor database. Nucleic Acids Res..

[31] Schommer C (2008). Control of jasmonate biosynthesis and senescence by miR319 targets. PLoS Biol..

[32] Donner TJ, Sherr I, Scarpella E (2009). Regulation of preprocambial cell state acquisition by auxin signaling in Arabidopsis leaves. Development.

[33] Pekker I, Alvarez JP, Eshed Y (2005). Auxin response factors mediate Arabidopsis organ asymmetry via modulation of KANADI activity. Plant Cell.

[34] Kalve S, De Vos D, Beemster GTS (2014). Leaf development: A cellular perspective. Front. Plant Sci..

[35] Chung Y (2019). Auxin response factors promote organogenesis by chromatin-mediated repression of the pluripotency gene SHOOTMERISTEMLESS. Nat. Commun..

[36] Hake S (2004). The role of Knox genes in plant development. Annu. Rev. Cell Dev. Biol..

[37] Ragni L, Belles-Boix E, Günl M, Pautot V (2008). Interaction of KNAT6 and KNAT2 with Brevipedicellus and Pennywise in Arabidopsis inflorescences. Plant Cell.

[38] Jofuku KD, Den Boer BGW, Van Montagu M, Okamuro JK (1994). Control of arabidopsis flower and seed development by the homeotic gene APETALA2. Plant Cell.

[39] Würschum T, Groß-Hardt R, Laux T (2006). APETALA2 regulates the stem cell niche in the Arabidopsis shoot meristem. Plant Cell.

[40] Kiferle C (2015). Tomato R2R3-MYB proteins SlANT1 and SlAN2: Same protein activity, different roles. PLoS ONE.

[41] van der Knaap E (2014). What lies beyond the eye: The molecular mechanisms regulating tomato fruit weight and shape. Front. Plant Sci..

[42] Rodríguez-Leal D, Lemmon ZH, Man J, Bartlett ME, Lippman ZB (2017). Engineering quantitative trait variation for crop improvement by genome editing. Cell.

[43] Sato S (2012). The tomato genome sequence provides insights into fleshy fruit evolution. Nature.

[44] Lemmon ZH (2016). The evolution of inflorescence diversity in the nightshades and heterochrony during meristem maturation. Genome Res..

[45] Train CM, Glover NM, Gonnet GH, Altenhoff AM, Dessimoz C (2017). Orthologous Matrix (OMA) algorithm 2.0: More robust to asymmetric evolutionary rates and more scalable hierarchical orthologous group inference. Bioinformatics.

[46] Kanehisa M, Goto S (2000). KEGG: Kyoto Encyclopedia of Genes and Genomes. Nucleic Acids Res..

[47] Ronen G, Carmel-Goren L, Zamir D, Hirschberg J (2000). An alternative pathway to β-carotene formation in plant chromoplasts discovered by map-based cloning of Beta and old-gold color mutations in tomato. Proc. Natl. Acad. Sci. USA.

[48] Galpaz N, Ronen G, Khalfa Z, Zamir D, Hirschberg J (2006). A chromoplast-specific carotenoid biosynthesis pathway is revealed by cloning of the tomato white-flower locus. Plant Cell.

[49] Wang S (2017). Identification of anthocyanin composition and functional analysis of an anthocyanin activator in *Solanum nigrum* fruits. Molecules.

[50] Colliver S (2002). Improving the nutritional content of tomatoes through reprogramming their flavonoid biosynthetic pathway. Phytochem. Rev..

[51] Gao Y (2018). Tomato SlAN11 regulates flavonoid biosynthesis and seed dormancy by interaction with bHLH proteins but not with MYB proteins. Hortic. Res..

[52] Khusnutdinov E, Sukhareva A, Panfilova M, Mikhaylova E (2021). Anthocyanin biosynthesis genes as model genes for genome editing in plants. Int. J. Mol. Sci..

[53] Beckles DM, Hong N, Stamova L, Luengwilai K (2012). Biochemical factors contributing to tomato fruit sugar content: A review. Fruits.

[54] Beauvoit BP (2014). Model-assisted analysis of sugar metabolism throughout tomato fruit development reveals enzyme and carrier properties in relation to vacuole expansion. Plant Cell.

[55] Yeager AF (1927). Determinate growth in the tomato. J. Hered..

[56] Xu C (2015). A cascade of arabinosyltransferases controls shoot meristem size in tomato. Nat. Genet..

[57] Hay A, Tsiantis M (2010). KNOX genes: Versatile regulators of plant development and diversity. Development.

[58] Ori N (2007). Regulation of LANCEOLATE by miR319 is required for compound-leaf development in tomato. Nat. Genet..

[59] Ben-Gera H, Ori N (2012). Auxin and LANCEOLATE affect leaf shape in tomato via different developmental processes. Plant Signal. Behav..

[60] Yanai O, Shani E, Russ D, Ori N (2011). Gibberellin partly mediates LANCEOLATE activity in tomato. Plant J..

[61] Ben-Gera H (2012). ENTIRE and GOBLET promote leaflet development in tomato by modulating auxin response. Plant J..

[62] Cárdenas PD (2016). GAME9 regulates the biosynthesis of steroidal alkaloids and upstream isoprenoids in the plant mevalonate pathway. Nat. Commun..

[63] Itkin M (2013). Biosynthesis of antinutritional alkaloids in solanaceous crops is mediated by clustered genes. Science (80-).

[64] Thouet J, Quinet M, Ormenese S, Kinet JM, Périlleux C (2008). Revisiting the involvement of Self-pruning in the sympodial growth of tomato. Plant Physiol..

[65] Klee HJ, Giovannoni JJ (2011). Genetics and control of tomato fruit ripening and quality attributes. Annu. Rev. Genet..

[66] Vallarino JG (2017). Postharvest changes in LIN5-down-regulated plants suggest a role for sugar deficiency in cuticle metabolism during ripening. Phytochemistry.

[67] Bolger AM, Lohse M, Usadel B (2014). Trimmomatic: A flexible trimmer for Illumina sequence data. Bioinformatics.

[68] Haas BJ (2013). De novo transcript sequence reconstruction from RNA-seq using the Trinity platform for reference generation and analysis. Nat. Protoc..

[69] Li W, Godzik A (2006). Cd-hit: A fast program for clustering and comparing large sets of protein or nucleotide sequences. Bioinformatics.

[70] Langmead B, Trapnell C, Pop M, Salzberg SL (2009). Ultrafast and memory-efficient alignment of short DNA sequences to the human genome. Genome Biol..

[71] Li B, Dewey CN (2011). RSEM: Accurate transcript quantification from RNA-Seq data with or without a reference genome. BMC Bioinform..

[72] Jones P (2014). InterProScan 5: Genome-scale protein function classification. Bioinformatics.

[73] Kumar L, Futschik ME (2007). Mfuzz: A software package for soft clustering of microarray data. Bioinformation.

[74] Kumar S, Stecher G, Li M, Knyaz C, Tamura K (2018). MEGA X: Molecular evolutionary genetics analysis across computing platforms. Mol. Biol. Evol..

[75] Schmittgen TD, Livak KJ (2008). Analyzing real-time PCR data by the comparative CT method. Nat. Protoc..

[76] Chu H (2013). Expression of the sweetpotato R2R3-type IbMYB1a gene induces anthocyanin accumulation in Arabidopsis. Physiol. Plant..

[77] Yoo HJ (2017). Inferring the genetic determinants of fruit colors in tomato by carotenoid profiling. Molecules.

[78] Gupta P, Sreelakshmi Y, Sharma R (2015). A rapid and sensitive method for determination of carotenoids in plant tissues by high performance liquid chromatography. Plant Methods.

[79] Park S (2020). Rapid generation of transgenic and gene-edited *Solanum nigrum* plants using Agrobacterium-mediated transformation. Plant Biotechnol. Rep..

[80] Werner S, Engler C, Weber E, Gruetzner R, Marillonnet S (2012). Fast track assembly of multigene constructs using golden gate cloning and the MoClo system. Bioeng. Bugs.

[81] Van Eck J, Keen P, Tjahjadi M (2019). Agrobacterium tumefaciens-mediated transformation of tomato. Methods Mol. Biol..

