# Supplementary material for: The comparisons of expression pattern reveal molecular regulation of fruit metabolites in S. nigrum and S. lycopersicum
Source: Sci Rep. 2022 Mar 23;12:5001. doi: 10.1038/s41598-022-09032-z (PMC8943121; doi:10.1038/s41598-022-09032-z)
Supplement: Supplementary file 1 — Supplementary Information 1. [file 41598_2022_9032_MOESM1_ESM.pdf]

Supplementary Information for

The comparisons of expression pattern reveal molecular regulation of fruit metabolites in *S. nigrum* and *S. lycopersicum*

Jung Heo†, Woo Young Bang†, Jae Cheol Jeong, Sung-Chul Park, Je Min Lee, Sungho Choi, Byounghee Lee, Young Koung Lee, Keunhwa Kim* and Soon Ju Park*

†J.H. and W.Y.B. contributed equally to this work.

*Correspondence should be addressed to K.K. (gonna04@wku.ac.kr) and S.J.P. (sjpark75@wku.ac.kr)

This PDF includes

Supplementary Figure S1–7 with legends

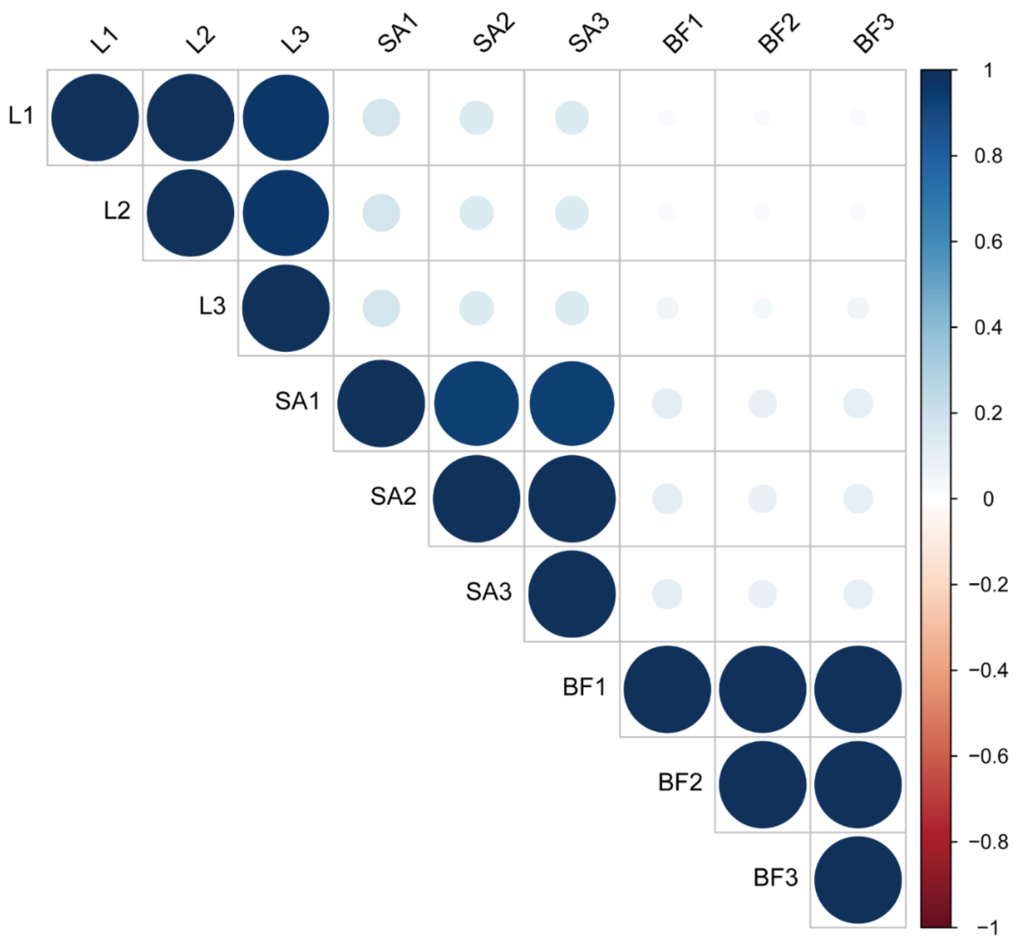

Supplementary Figure S1. Expressional correlation between *S. nigrum* samples.

The size and color of circles indicate the level of expressional correlation (Pearson's correlation coefficient). L, leaf; SA, shoot apex; BF, black fruit.

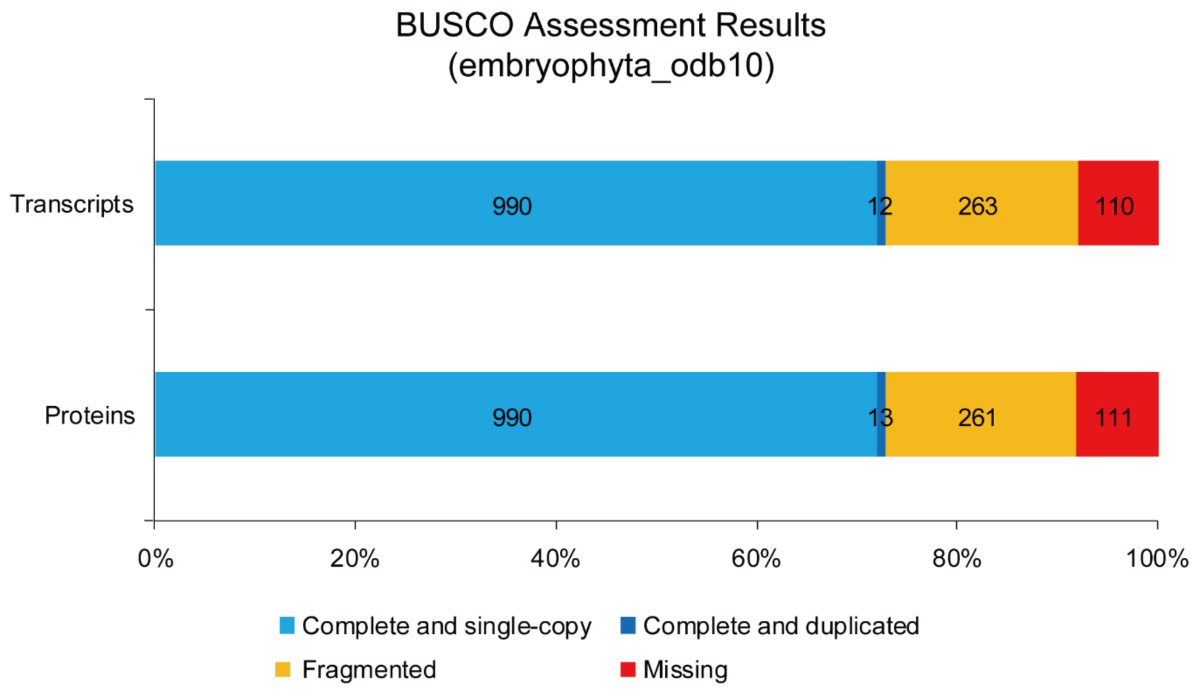

Supplementary Figure S2. BUSCO result of *S. nigrum* unigenes.

The BUSCO analysis was assessed using the embryophyta (version, odb10) lineage dataset. Each number in the bars indicates the number of each BUSCO.

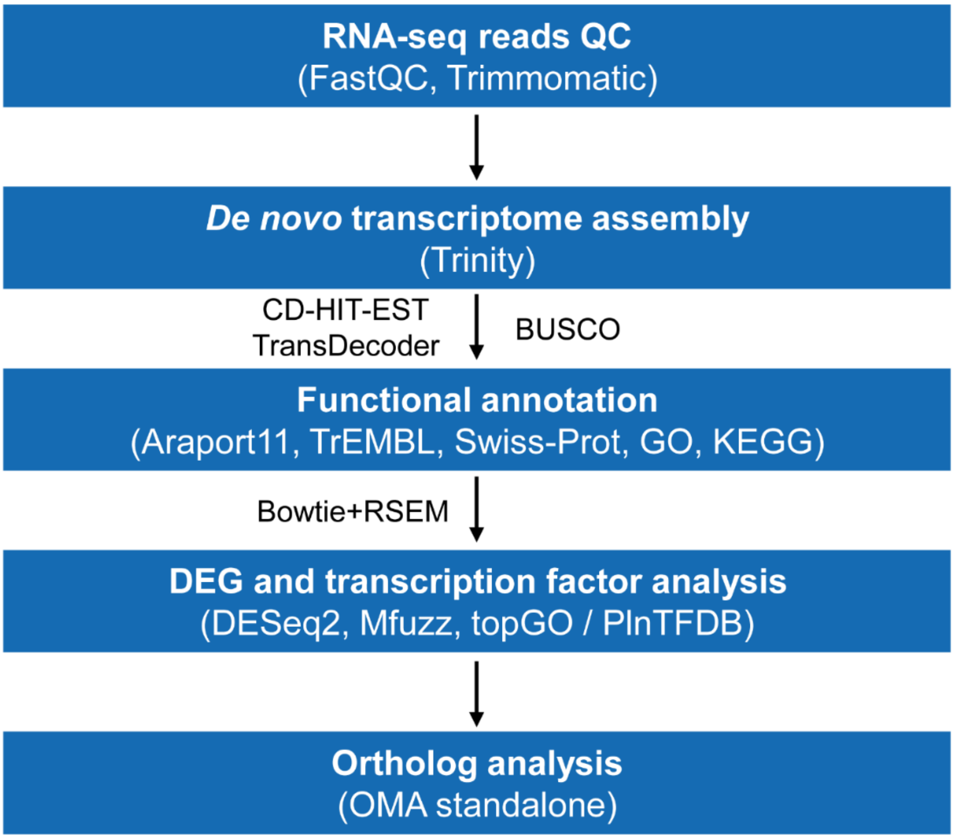

Supplementary Figure S3. Workflow of *S. nigrum* transcriptome analysis.

Bold white characters indicate each step of the transcriptome analysis. Other characters represent used tools in each step.

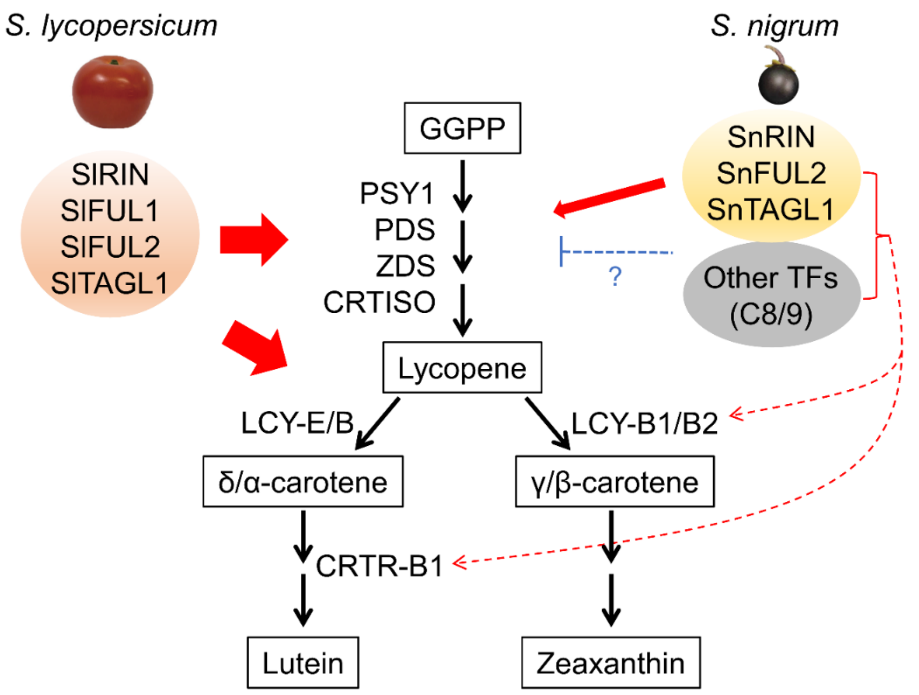

Supplementary Figure S4. A hypothetical model for the molecular regulation of carotenoid biosynthesis in the fruits of *S. lycopersicum* and *S. nigrum*.

Gene expressions of the ripening regulators such as SlRIN, SlFUL1/2 and SlTAGL1 are upregulated in the fruits of *S. lycopersicum*, which induce the transcription of various carotenoid biosynthetic enzyme genes (red arrows on the left). Thus, many kinds of carotenoids are accumulated in tomato fruit. On the other hand, in the fruits of *S. nigrum*, there might be an antagonistic regulation for carotenoid biosynthetic enzyme genes against the positive regulation of SnRIN, SnFUL2, and SnTAGL1, possibly through other BF-enriched TFs (red arrow and blue dashed-line on the right). Alternatively, certain enzyme genes such as LCY-B1/B2 and CRTR-B1 are upregulated by the combined actions of BF-enriched TFs, which might cause selective accumulations of β-carotene and lutein in the fruits of *S. nigrum* (red dashed-arrows on the right).

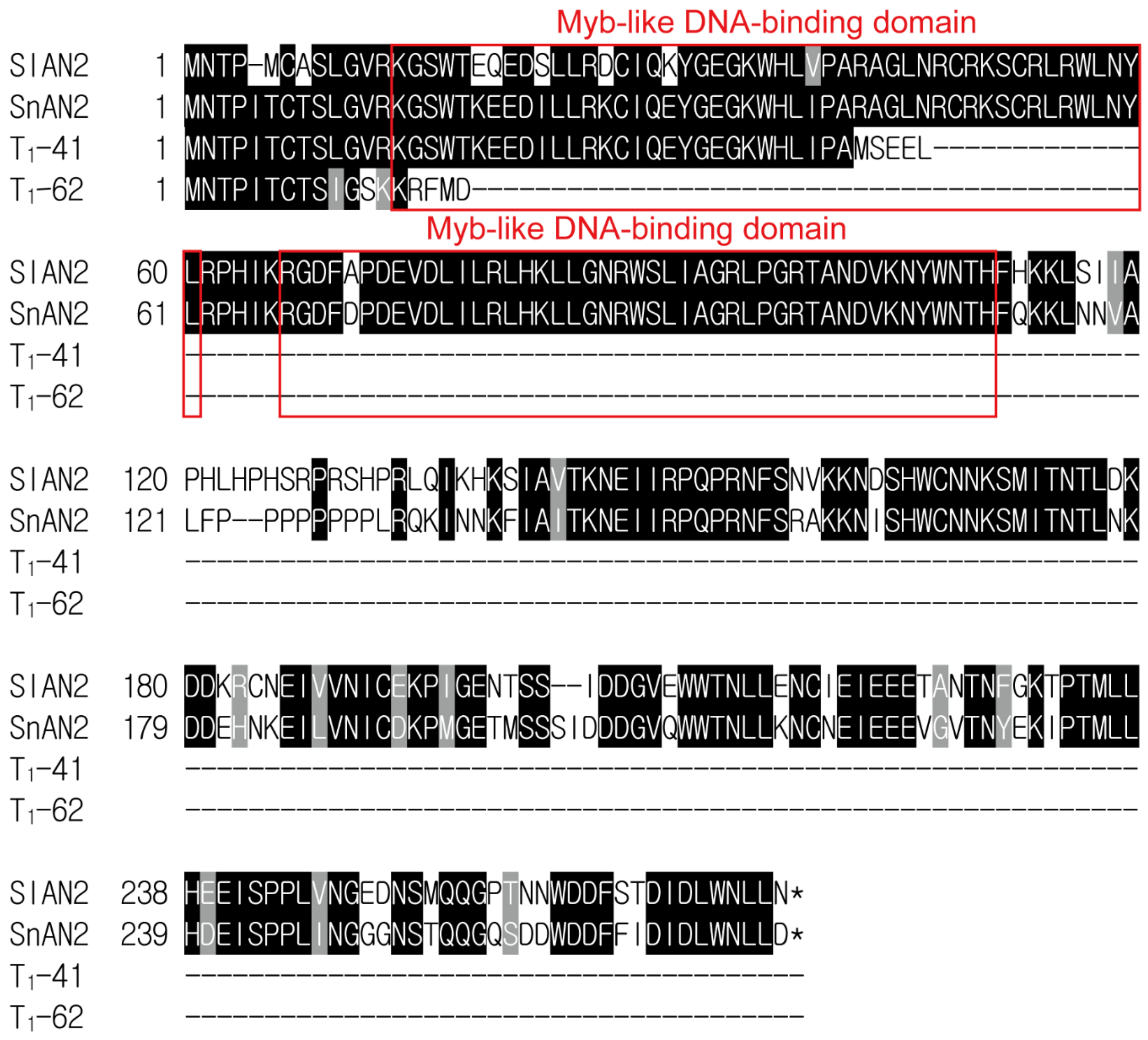

Supplementary Figure S5. Protein alignment in SlAN2, SnAN2, and *snan2-cr*.

All protein sequences were aligned using Clustal Omega and two domains were predicted based on Pfam. T_1-41 and 62, two independent *snan2-cr* mutants in T_1 generation.

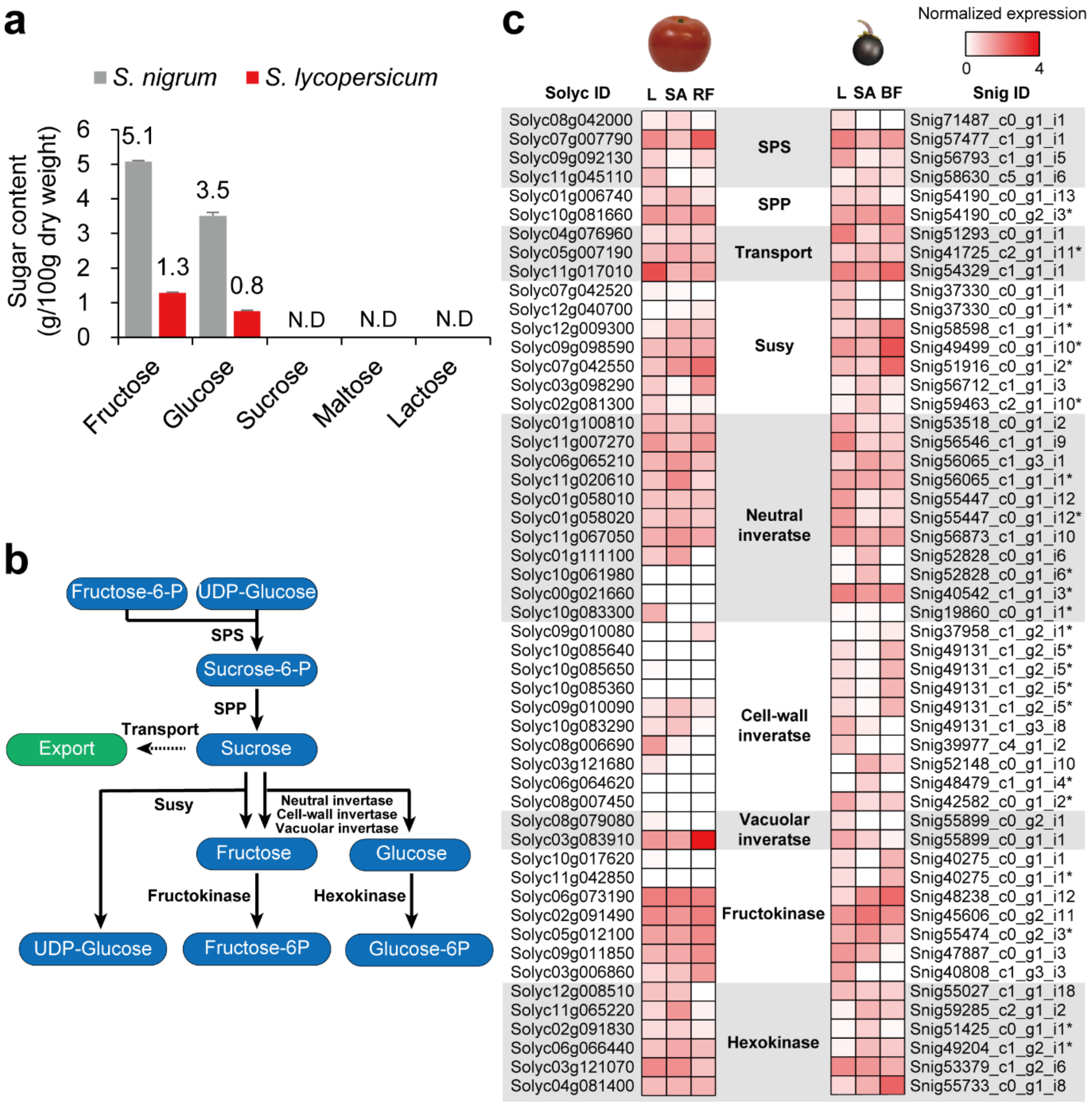

Supplementary Figure S6. Comparison of metabolite and expression profiles in the sugar metabolism pathway.

(a) Sugar contents in mature fruits in *S. nigrum* and *S. lycopersicum*. Data are shown as mean ± standard deviation: n=4, a minimum of 50 fruits were pooled and measured with two technical replicates. N.D, not detected. **(b)** Sugar metabolism pathway. Dotted arrow indicates the condensed pathway. **(c)** Expression profiles of genes regulating sugar metabolism. *S. nigrum* unigenes with asterisk represent genes with highest homology. The expression was normalized by log10(TPM+1). L, leaf; SA, shoot apex; RF, red fruit; BF, black fruit.

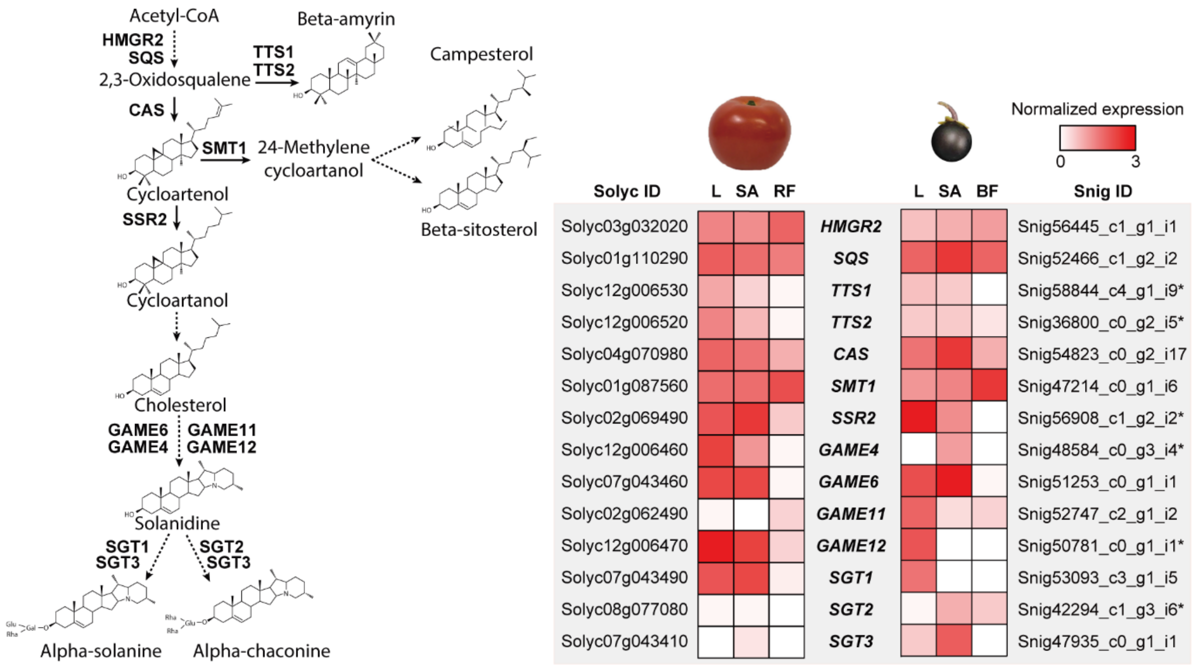

Supplementary Figure S7. Comparison of expression profiles in steroidal glycoalkaloid (SGA) biosynthesis pathway.

SGA biosynthesis pathway and expression profiles of SGA biosynthetic genes. Dotted arrow indicates the condensed pathway. *S. nigrum* unigenes with asterisk represent genes with highest homology. The expression was normalized by $\log_{10}$(TPM+1). L, leaf; SA, shoot apex; RF, red fruit; BF, black fruit.
